# Application of Protein-Protein Interaction Network Analysis in Order to Identify Cervical Cancer miRNA and mRNA Biomarkers

**DOI:** 10.1155/2023/6626279

**Published:** 2023-09-14

**Authors:** Parinaz Tabrizi-Nezhadi, Habib MotieGhader, Masoud Maleki, Soner Sahin, Sajjad Nematzadeh, Mahsa Torkamanian-Afshar

**Affiliations:** ^1^Department of Biology, Tabriz Branch, Islamic Azad University, Tabriz, Iran; ^2^Department of Health Ecosystem, Medical Faculty, Nisantasi University, Istanbul, Turkey; ^3^Software Engineering Department, Engineering Faculty, Topkapi University, Istanbul, Turkey; ^4^Department of Computer Engineering, Faculty of Engineering and Architecture, Nisantasi University, Istanbul, Turkey

## Abstract

Cervical cancer (CC) is one of the world's most common and severe cancers. This cancer includes two histological types: squamous cell carcinoma (SCC) and adenocarcinoma (ADC). The current study aims at identifying novel potential candidate mRNA and miRNA biomarkers for SCC based on a protein-protein interaction (PPI) and miRNA-mRNA network analysis. The current project utilized a transcriptome profile for normal and SCC samples. First, the PPI network was constructed for the 1335 DEGs, and then, a significant gene module was extracted from the PPI network. Next, a list of miRNAs targeting module's genes was collected from the experimentally validated databases, and a miRNA-mRNA regulatory network was formed. After network analysis, four driver genes were selected from the module's genes including *MCM2*, *MCM10*, *POLA1*, and *TONSL* and introduced as potential candidate biomarkers for SCC. In addition, two hub miRNAs, including miR-193b-3p and miR-615-3p, were selected from the miRNA-mRNA regulatory network and reported as possible candidate biomarkers. In summary, six potential candidate RNA-based biomarkers consist of four genes containing MCM2, MCM10, POLA1, and TONSL, and two miRNAs containing miR-193b-3p and miR-615-3p are opposed as potential candidate biomarkers for CC.

## 1. Introduction

In developing countries, cervical cancer (CC) is the second most common cancer [1]. This cancer is a type of cancer that occurs in the cervix cells [2]. In other words, the growth of abnormal cells in the lining of the cervix is called CC. The CC includes two common histological types: squamous cell carcinoma (SCC) and adenocarcinoma (ADC) [3]. In this study, SCC has been studied. Squamous cell carcinoma is the most common CC, accounting for 70% of cases.

Cancer biomarker detection is one of the crucial challenges in cancer studies. A cancer biomarker is a biological molecule showing cancer's presence [4]. Different types of cancer biomarkers include molecular, radiographic, histologic, and physiologic [5]. The goal of this study was to identify molecular biomarkers for CC. The molecular samples which have been analyzed in this study are transcriptome profiles.

Different studies have been conducted in order to discover RNA-based cancer biomarkers. Wen et al. [6] introduced miRNA-873-5p as a potential biomarker and promising therapeutic approach for CC in their research. Cao et al. [7] reported CCAT2 as a candidate biomarker for diagnosing and prognostic predicting CC. In another study, EFNA1 was introduced as a novel prognostic biomarker for CC by Shen and colleagues. Moreover, Anja Nilsen and colleagues [8] proposed miR-200a, miR-200b, and miR-429 as candidate biomarkers in CC. Moreover, Zhao et al. [9] concluded that SPP1 could be a prognostic biomarker in CC. In another study, INHBA was introduced as a prognostic biomarker. Also, authors reported that this gene correlates with immune cell infiltration in CC [10]. Besides, Xinyang Zhang and colleagues introduced a circular RNA named circYPEL2 as a potential biomarker for clinical research of CC.

Moreover, some network-based studies have been conducted to identify cancer biomarkers. Guo and colleagues [11] proposed a network-based algorithm to identify cancer biomarkers. In 2021, Hua et al. introduced a gene coexpression network to identify the biomarkers in human tumors [12]. In another study, Zhang and colleagues [13] proposed two lncRNAs as prognostic biomarkers in gastric cancer based on an integrated analysis of the lncRNA-associated ceRNA network. Wang and colleagues [14] proposed a deep learning model and similarity network fusion to recognize biomarkers through multiomics data analysis in prostate cancer. Tong et al. [15] performed theoretical and in silico analyses and proposed MYC as a dynamic network biomarker in colon and rectal cancer. In addition, Li and colleagues [16] proposed a competing endogenous network for the identification of prognostic biomarkers in bladder cancer. In 2020, Yang et al. introduced a gene regulation network analysis and proposed YAP1 as a prognostic biomarker in pancreatic cancer.

Proteins control biological processes, molecular functions, and cellular mechanisms and determine disease and healthy states [16]. Therefore, the study of proteins' interactions inside the cell is critical. Thus, a protein-protein interaction (PPI) network analysis was studied in the current project.

miRNAs are small noncoding RNA molecules that regulate mRNAs from being translated [17, 18]. This type of RNA regulates gene expression at the posttranscriptional level and can be found in tissue, blood, and body fluids [19]. Recently, miRNAs have been introduced as prognostic and diagnostic biomarkers in different breast, colorectal, ovarian, and cervical cancers. The interaction between miRNAs and genes shows a regulatory relationship between miRNAs and genes [20]. In this regard, different cancer studies have investigated miRNA-mRNA interaction networks. Negar and colleagues [21] introduced a miRNA-mRNA network-based biomarker for Alzheimer's disease. Besides, Motieghader et al. [22] proposed a miRNA-mRNA module prognostic biomarker for the early detection of colorectal cancer based on coexpression network analysis. Moreover, Adhami and colleagues [23] proposed a miRNA-mRNA subnetwork as a prognostic biomarker for breast cancer subtype stratification. In this project, interactions of miRNAs and target genes have been studied and two significant miRNAs have been introduced as prognostic biomarkers for cervical cancer.

Cancer driver genes are the genes in which mutations in these genes cause tumor growth [24]. These genes can be of two types: tumor suppressor genes and proto-oncogenes. DriverDBv3 [25] is an online database containing human cancer driver genes with mutation, CNV, and methylation information. In the current project, a list of driver genes for cervical cancer was collected from this database.

**Figure 1:**
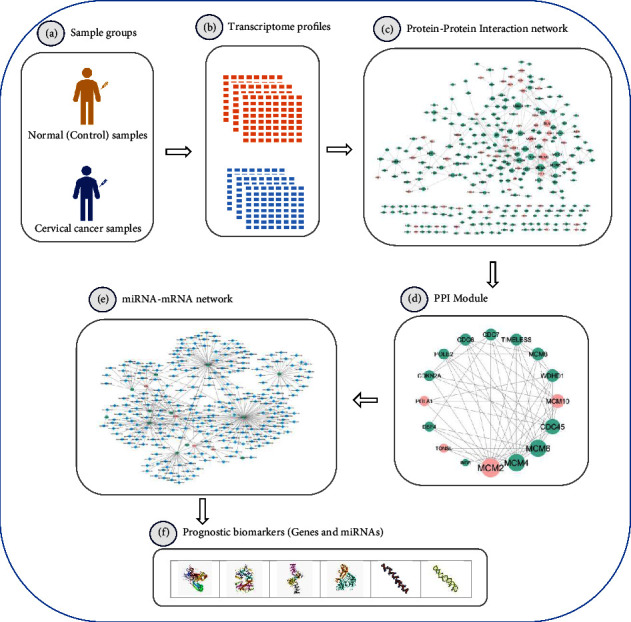
The workflow diagram of the project. In this project, a protein-protein interaction (PPI) network analysis and miRNA-mRNA regulatory network were utilized to discover RNA-based biomarkers in cervical cancer. (a, b) First, a transcriptome dataset for normal and CC samples was gathered from the GEO database with the accession number GSE63514. (c) Next, a PPI network was constructed for the DEG (*p*_value <0.05) between normal and CC groups thanks to the STRING database. (d) Then, a significant protein module containing 16 proteins was extracted from the PPI network. (e) Subsequently, a miRNA-mRNA regulatory network was reconstructed. (f) Consequently, four cancer driver genes (*MCM2*, *MCM10*, *POLA1*, and *TONSL*) and two miRNAs (*miR-193b-3p* and *miR-615-3p*) were introduced as potential candidate biomarkers for CC patients.

The current study aimed at identifying the genes and miRNAs as prognostic biomarkers in CC. In this regard, this project used a PPI network analysis to discover candidate prognostic biomarkers for CC. In this project, a normal and CC samples' transcriptome profile was first downloaded from the NCBI-GEO with accession number GSE63514. Then, differentially expressed genes (DEGs) between normal and cervical cancer groups were calculated, and a list of significant genes was selected for network construction. Next, a PPI network was constructed for the selected genes in the STRING [26] online tool. After that, a significant protein module was extracted from the PPI network. Subsequently, the miRNAs targeting module's genes were collected from the miRTarBase [27] online database. Consequently, four driver genes (MCM2, MCM10, POLA1, and TONSL) and two miRNAs (miR-193b-3p and miR-615-3p) were introduced as prognostic biomarkers in CC. The workflow diagram of this project is depicted in Figure 1.

## 2. Materials and Methods

### 2.1. Dataset and Preprocessing

In this project, the gene expression profile with accession number GSE63514 was downloaded from the NCBI-GEO. These data include 24 normal and 28 cervical SCC samples from the tissue specimens. An annotation file with accession number GPL570 was used to assign probes to gene IDs.

In this study, a protein-protein interaction (PPI) network-based approach was applied to discover cancer driver genes as prognostic biomarkers for CC patients. To this end, at first, differentially expressed genes (DEGs) between normal and CC groups were calculated using the *Bonferroni* method. The genes with *p*_value <0.05 were selected and assumed as the primary gene list. This primary gene list contains 1335 genes (Supplementary file S1). Then, a PPI network was reconstructed for the primary gene list, thanks to the STRING [26] online tool.

### 2.2. PPI Network Reconstruction and Module Extraction

After calculating DEGs, the primary gene list, including 1335 genes, was imported to the STRING online database. Then, a PPI network was constructed for the primary gene list with the following parameters. *Network type: physical subnetwork, active interaction sources: experiments,* and *Minimum required interaction score: medium confidence (0.400)*.

Cytoscape software [28] version 3.8.2 was utilized to evaluate and analyze the PPI network. In order to discover highly interacted proteins in the PPI network (PPI modules), the ClusterViz [29] plugin was applied. ClusterViz is a Cytoscape plugin that discovers modules in a biological network using three different clustering algorithms, including FAG-EC, MCODE, and EAGLE. In the current project, FAG-EC with complex threshold = 10 was used for detecting PPI modules.

### 2.3. Cervical Cancer Driver Genes

DriverDBv3 [25] (a database for human cancer driver gene research) is a database that contains cancer driver genes for different cancers. In this database, a list of cancer driver genes was defined by bioinformatics tools in multiple features, including mutation, CNV, and methylation drivers. After gathering all of the cancer driver genes from this database, only CC driver genes were maintained. In total, 1865 driver genes were obtained for CC (Supplementary files S2).

### 2.4. miRNA-mRNA Regulatory Network

The goal of this section is to analyze interactions between miRNAs and mRNAs. miRNAs play important roles in development and tumorigenesis by targeting tumor suppressor genes or oncogenes. One gene can be regulated by multiple miRNAs and one miRNA can regulate multiple genes [30]. To do this, a miRNA-mRNA regulatory network was constructed. In order to collect the list of miRNA target genes, the miRTarBase [27] online database was used. miRTarBase is a microRNA-target interaction database that brings regulatory information using experimentally validated methods.

### 2.5. Enrichment Analysis

In order to identify biological processes, molecular functions, and cellular components of the module's genes, the GeneCodis [31] online tool was utilized to identify biological processes. Also, pathway enrichment analysis was carried out using this tool based on the Reactome [32] database.

TAM [33] is an online database for identifying the miRNA family. This database was applied to determine the miRNA family. All miRNAs in the miRNA-mRNA regulatory network were imported to the TAM database, and significant miRNA families were reported.

## 3. Results

### 3.1. Protein-Protein Interaction (PPI) Network Analysis

**Figure 2:**
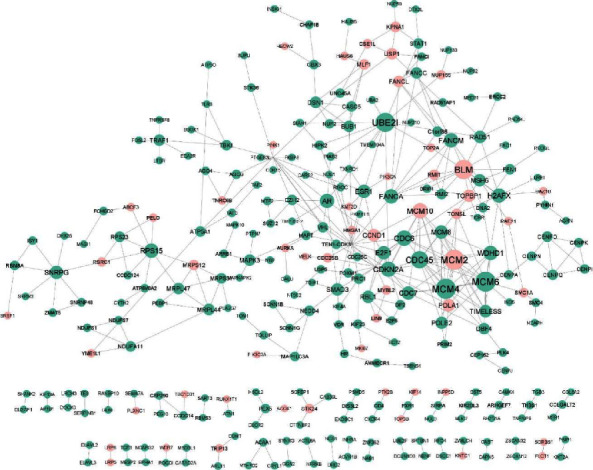
PPI network for the primary gene list. The degree of the nodes indicates their interactions. This network has 290 nodes (proteins) and 381 edges (interactions). CDK1 has the highest interactions in the network. Green nodes indicate regular ones, and red nodes indicate cervical cancer driver genes based on the DriverDBv3 [25] report.

At first, 1335 differentially expressed genes (DEGs) between normal and cervical cancer groups with adjusted *p*_values smaller than 0.05 were selected and assumed as the primary gene list. Then, a PPI network was constructed for these genes, thanks to the STRING [26] online database. Of 1335 genes, 1045 were disconnected in the constructed PPI network. Therefore, these disconnected genes were removed from the PPI network. After removing these disconnected genes from the network, 290 genes remained and were assumed as the primary gene list.  Figure 2 shows the constructed PPI network for these remaining genes. This figure demonstrates that regular and cervical cancer driver genes are indicated with green and red colors, respectively.

### 3.2. Module Extraction

**Figure 3:**
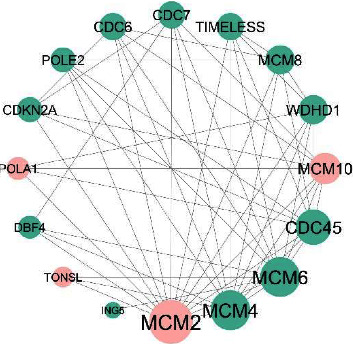
The significant protein module extracted from the PPI network. Green and red color nodes indicate regular and driver genes, respectively.

After constructing the PPI network in the STRING database, the network was imported into the Cytoscape [28] software package. After analyzing the network using this software, 290 proteins and 381 interactions were observed. Then, thanks to the Clusterviz [29] plugin, a significant module including 16 proteins was extracted from the constructed PPI network. This module is shown in Figure 3. In this module, red nodes indicate cervical cancer driver genes. This module's four proteins, MCM2, MCM10, POLA1, and TONSL, are cervical cancer driver genes based on the DriverDBv3 [25] report. These genes can be assumed as potential candidate biomarkers in cervical cancer. The logFC values of the *MCM2*, *MCM10*, *POLA1*, and *TONSL* in cervical cancer versus normal groups are 2.3, 3.37, 1.76, and 0.587, respectively. The results show that these four genes have a higher expression in cervical cancer samples than in normal samples.

#### 3.2.1. miRNA-mRNA Regulatory Network

**Figure 4:**
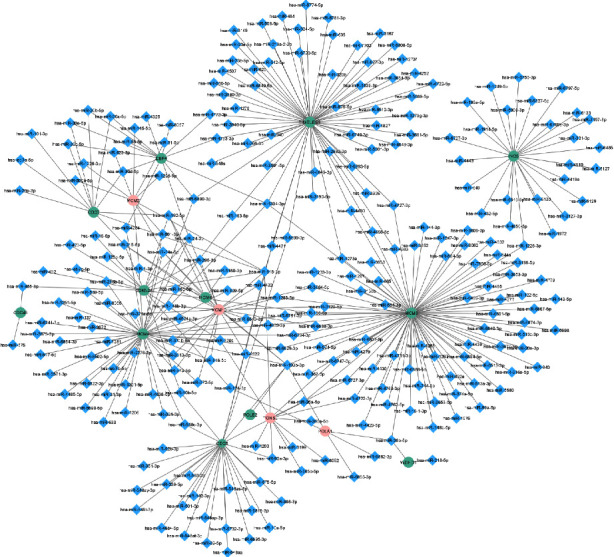
miRNA-mRNA regulatory network. Circle and diamond nodes indicate genes and miRNAs, respectively. As well as, red shapes indicate cancer driver genes. High-degree miRNAs are miR-193b-3p and miR-615-3p. This network contains 262 miRNAs and 16 target genes.

miRTarBase [27], an online database, was utilized to identify a list of miRNAs targeting module's genes. All 16 genes of the module were imported into the miRTarBase database, and 262 miRNAs were found.  Figure 4 depicts the miRNA-mRNA regulatory network. In this network, *miR-193b-3p* and *miR-615-3p* are high-degree miRNAs. This means that these miRNAs regulate seven genes of the PPI module and can play a significant role in regulating the PPI module's genes. Complete interaction information of this network is reported in Supplementary file S3. Also, seven hub miRNAs (high-degree miRNAs) of the miRNA-mRNA network are reported in Table 1. Two hub miRNAs, including *miR-193b-3p* and *miR-615-3p*, have been proposed as potential candidate biomarkers for CC patients.

#### 3.2.2. Enrichment Analysis of Genes

Gene Ontology (GO) and pathway enrichment analysis were performed for the extracted module thanks to the GeneCodis [31] online tool. The results show that this module significantly enriched in “*Activation of the prereplicative complex*” biological process, “*MCM complex*” cellular component, and “*DNA replication origin binding*” molecular function. Moreover, this module was significantly enriched in “*Activation of the prereplicative complex*,” “*Mitotic G1 phase and G1/S transition*,” and “*Activation of ATR in response to replication stress*” pathways. The GO and pathway enrichment analysis details are reported in Supplementary file S4.

#### 3.2.3. Enrichment Analysis of miRNAs

TAM [33] is an online tool for miRNA set analysis. All 262 miRNAs from the miRNA-mRNA regulatory network were imported to the TAM tool, and significant miRNA families were obtained to conduct miRNA enrichment analysis. The results show that these miRNAs are significantly enriched in *mir-30*, *mir-10*, *mir-124*, *mir-26*, *mir-290*, and *let-7* families (see Supplementary file S4).

## 4. Discussion

This project used a PPI and miRNA-mRNA network analysis to identify potential candidate biomarkers for SCC patients. To this end, at first, DEGs with adjusted *p*_value <0.05 between normal and SCC groups were selected. Then, a PPI network was constructed for DEGs in the STRING online database. After constructing the PPI network, one significant module containing 16 genes was extracted from the PPI network. Out of 16 genes in the modules, four of them, including *MCM2*, *MCM10*, *POLA1*, and *TONSL*, are SCC driver genes. In the next step, a list of miRNAs targeting module's genes was collected from the miRTarBase online database, and a miRNA-mRNA regulatory network was drawn. The constructed miRNA-mRNA network contains 262 miRNAs and 16 target genes.

Gene Ontology and a pathway enrichment analysis were performed for the module's genes. The results show that these genes are most significantly enriched in the “Activation of the prereplicative complex” biological process, “MCM complex” cellular component, and “DNA replication origin binding” molecular function. These genes are most significantly enriched in the “Activation of the prereplicative complex” pathway. Moreover, the list of miRNAs in the miRNA-mRNA regulatory network was imported to the TAM [33] online database. The results show that these miRNAs are most significantly enriched in *mir-30*, *mir-10*, *mir-124*, *mir-26*, *mir-290*, and *let-7* families. Complete GO and pathway enrichment analysis information are available in Supplementary file S4.

Four driver genes from the extracted module, including *MCM2*, *MCM10*, *POLA1*, and *TONSL*, were selected and introduced as candidate gene biomarkers for CC patients. In addition, two hub miRNAs, including *miR-193b-3p* and *miR-615-3p*, were selected from the miRNA-mRNA interaction network and proposed as candidate miRNA biomarkers for CC patients.

Lu and colleagues [34] in 2021 utilized a bioinformatics analysis and suggested that MCM2 regulates CC progression. In another study, Kaur and colleagues [35] reported that the expression level of MCM2 was upregulated with increasing fold change during the progression from the low-grade squamous intraepithelial lesion to the high-grade squamous intraepithelial lesion and the highest in SCC. Furthermore, this gene has the most remarkable fold change in SCC compared to the normal cervix [35]. Moreover, Sérgio Amaro Filho and colleagues [36] revealed that an increased expression of MCM2 was found in invasive CC compared to controls.

Murayama et al. [37] reported that the expression level of MCM10 is upregulated in cancer stem-like cells. In another study, Yang and colleagues [38] introduced MCM10 as a potential diagnostic tool and a promising target for breast carcinoma. Besides, Mahadevappa et al. [39] reported that MCM10 plays a vital role in breast cancer progression, and this gene was introduced as a potential prognostic biomarker for breast cancer patients. Moreover, Cui and colleagues [40] revealed that MCM10 was significantly upregulated in prostate cancer. They suggested this gene as a potential diagnostic and therapeutic target for prostate cancer [40].

Liu and colleagues [41] performed a gene coexpression network analysis and introduced TIPIN and POLA1 as potential prognostic biomarkers for CC patients. In another study, Lijun Yu et al. [42] reported that the high expression of *POLA1*, *TOP2A*, and *RRM2* increased in the multistep of CC. Based on Yu et al. [42] reports, these genes may be targets for treating CC.

TONSL was introduced as an oncogene in esophageal, lung, and cervical cancers [43]. This gene is significantly upregulated in hepatocellular carcinoma tissues compared to normal liver tissues [43].

Huang and colleagues [44] revealed that the downregulation of miR-193b by targeting CCND1 promotes CC aggressiveness. m6A methylation regulates miRNA functions as a tumor suppressor in cervical tumors [44]. In a similar study, Han et al. [45] reported the impact of miR-193b-3p in CC and disclosed that NEAT1 could facilitate the radio-resistance of CC via binding miR-193b-3p. In another study, Jiménez-Wences and colleagues [46] revealed that the methylation levels of the miR-193b promoter were significantly lower in CC than in low-grade squamous intraepithelial lesion samples. Besides, this miRNA was introduced as a potential biomarker for CC based on a meta-analysis of transcriptomics data and network analysis [47]. Jing Feng and colleagues reported the effect of miR-615-3p on CC promotion [48]. Also, this miRNA's impact on different cancers was reported [49–52].

In conclusion, our results show that six RNAs including four genes (*MCM2*, *MCM10*, *POLA1*, and *TONSL*) and two miRNAs (*miR-193b-3p* and *miR-615-3p*) are proposed as candidate prognostic biomarkers for CC patients.

## 5. Conclusion

The current study uses a PPI network analysis to discover potential candidate driver gene biomarkers for SCC patients. To this end, the transcriptome profile of normal and SCC samples was first downloaded from the NCBI-GEO. Then, a PPI network was constructed, thanks to the STRING database. Next, after importing the constructed PPI network into Cytoscape software, a significant gene module with 16 genes was discovered. Of these 16 genes, 4 of them are SCC driver genes. After that, a list of miRNAs targeting module's genes was collected from the miRTarBase online database, and a miRNA-mRNA regulatory network was constructed. In the miRNA-mRNA network, two hub miRNAs (*miR-193b-3p* and *miR-615-3p*) were selected and introduced as potential biomarkers for SCC patients. These miRNAs regulate seven module genes and can be very important in SCC. Consequently, four driver genes of the module, including *MCM2*, *MCM10*, *POLA1*, and *TONSL*, and two hub miRNAs of the miRNA-mRNA network, including *miR-193b-3p* and *miR-615-3p*, are introduced as the potential candidate biomarkers for SCC patients, as well. Moreover, by gene set enrichment analysis, it seems that the genes of the module are most significantly enriched in the “Activation of the prereplicative complex” biological process. Also, miRNAs of the miRNA-mRNA network are significantly enriched in *mir-30*, *mir-10*, *mir-124*, *mir-26*, *mir-290*, and *let-7* families.

## Figures and Tables

**Table 1:** Seven hub miRNAs along with target genes in the miRNA-mRNA regulatory network.

miRNA	Degree	Target genes
miR-193b-3p	7	MCM8, MCM4, CDC6, MCM10, MCM6, POLA1, POLE2
miR-615-3p	7	TIMELESS, CDC6, MCM10, CDKN2A, TONSL, CDC7, MCM2
miR-34a-5p	5	MCM4, MCM10, CDKN2A, MCM2, MCM6C
miR-24-3p	4	MCM4, MCM10, CDKN2A, DBF4
miR-215-5p	4	MCM10, CDKN2A, CDC7, MCM6C
miR-192-5p	4	MCM10, CDKN2A, CDC7, MCM6C
miR-1304-3pc	4	MCM8, TIMELESS, MCM4, DBF4

## Data Availability

The datasets generated and analyzed in this study can be obtained from the corresponding author upon reasonable request. The datasets generated and analyzed during the current study are available in the GitHub repository, https://github.com/habibmoti/Cervical-Cancer.

## Supplementary Materials

Supplementary Materials This paper includes four supplementary files named S1 to S4.
