# Supplementary material for: Application of Protein-Protein Interaction Network Analysis in Order to Identify Cervical Cancer miRNA and mRNA Biomarkers
Source: ScientificWorldJournal. 2023 Sep 14;2023:6626279. doi: 10.1155/2023/6626279 (PMC10513823; doi:10.1155/2023/6626279)
Supplement: Supplementary Materials — This paper includes four supplementary files named S1 to S4. [file 6626279.f1.zip › Supplementary file S1 (1).pdf]

Primary gene list	Module's genes
A2ML1	TIMELESS
AANAT	CDKN2A
ABCF3	POLA1
ABHD1	ING5
ABHD10	MCM8
ABL2	MCM10
ACAA1	WDHD1
ACAN	MCM2
ACOT9	MCM6
ACOX1	MCM4
ACOX2	DBF4
ACPP	POLE2
ACSM1	CDC45
ACTL6A	CDC7
ACTN3	CDC6
ACTR3C	TONSL
ACVR1B	
ACY3	
ADAD1	
ADAM12	
ADAM22	
ADAM6	
ADAMTS16	
ADAMTS17	
ADARB2	
ADCY1	
ADH1B	
ADPGK	
AFF2	
AGAP3	
AGFG2	
AGO3	
AGO4	
AGPAT3	
AGRN	
AGTR2	
AHCYL2	
AHSP	
AIM2	
AKAP14	
ALAD	
ALPPL2	
AMMECR1	
ANGPTL6	
ANKRD27	
ANKRD34A	
ANKRD35	
ANKRD53	
ANO10	
AOC4P	
AP1B1	
AP5B1	
APCDD1L-AS1	
APOA5	

APOC1
APOC2
APOL5
AQP2
AR
ARHGAP10
ARHGAP35
ARHGAP6
ARHGEF10L
ARHGEF12
ARHGEF2
ARHGEF7
ARL11
ARL17B///ARL17A
ARL4D
ARRB1
ASAP1
ATAD2
ATN1
ATP2C1
ATP5A1
ATP5O
ATP6V0A2
AURKA
AXIN2
AZIN2
AZU1
B3GALT5
B4GALNT3
BAGE
BAZ1B
BBOX1
BCL2L2
BCL7A
BCL9
BEST1
BICDL2
BIVM
BLID
BLM
BMP8B
BMPR2
BORCS6
BRIP1
BRSK2
BSPRY
BTG2
BUB1
C12orf76
C15orf38-AP3S2///AP3S2
C15orf52
C15orf54
C15orf59
C16orf72
C16orf86
C17orf78

C18orf54
C1orf100
C1orf112
C20orf181
C20orf202
C21orf58
C2orf54
C2orf70
C3orf52
C3orf70
C4orf22
C4orf3
C5AR2
C5orf34
C5orf47
C6orf1
C9orf41-AS1
CA9
CAB39L
CABP1
CACNG7
CADM2
CALU
CAMK4
CAPN5
CARD11
CARD19
CARS2
CASD1
CASKIN1
CASR
CAST
CATSPERB
CATSPERG
CAV3
CBX3
CCDC124
CCDC14
CCDC172
CCDC59
CCDC61
CCDC88A
CCND1
CCSAP
CD163
CD24
CD4
CDC25B
CDC25C
CDC45
CDC6
CDC7
CDCA2
CDK1
CDK11A///CDK11B
CDK14

CDK15
CDK2
CDKN2A
CEACAM5
CELSR3
CENPA
CENPI
CENPJ
CENPK
CENPN
CENPO
CENPQ
CEP152
CEP290
CER1
CERCAM
CFC1B
CFD
CH17-360D5.1///NPY4R
CHAF1B
CHD2
CHDH
CHM
CHMP6
CHP1
CHRNA3
CHRNB4
CLCA4
CLCN1
CLDN19
CLEC7A
CLIC3
CLSPN
CNIH2
CNTLN
COA3
COL4A3
COL6A1
COL8A2
COLGALT2
COMMD6
COMT
CPA5
CPNE6
CPNE7
CPXCR1
CPXM2
CRABP2
CRB1
CREB3L3
CRHBP
CRISP2
CRISP3
CRLS1
CRNN
CROCCP2

CRYL1
CSE1L
CSF2RA
CSRP3
CST5
CTHRC1
CTNNA3
CTRB2///CTRB1
CTTNBP2
CTXN3
CUX1
CXCR2
CXCR4
CYMP
CYP1A2
CYP27B1
CYP2A6
CYP2C9
CYP2F1
CYP3A4
CYP3A43
CYP3A5
CYP4A22///CYP4A11
CYP4F22
CYP51A1-AS1
CYSRT1
CYTH2
DBF4
DCLRE1B
DCST1
DCUN1D3
DDIAS
DDO
DECR2
DEK
DEPDC1
DGCR2
DHX35
DICER1-AS1
DIS3L2
DISP3
DKFZP434A062
DKFZP434K028
DKFZp451B082
DLG3-AS1
DLGAP1
DNA2
DNAH9
DNAJC30
DNMBP
DOC2A
DOCK6
DOK4
DONSON
DPP4
DPP6

DRC3
DRD2
DSCC1
DSN1
DSTYK
DTL
DTX3
DTX3L
DUOX1
DXO
DYNC2LI1
E2F1
E2F5
ECT2
EDA2R
EDN3
EEF2KMT
EGFEM1P
EGFLAM-AS2
EGFLAM-AS4
EHD3
EI24
EIF2AK3
EIF4E
ELAVL2
ELAVL3
EML3
EMX1
ENDOU
ENSA
ENTPD6
EPB41L1
EPHA1
EPHX2
EPS8L1
EPS8L2
ERCC2
ESCO2
ESR1
ETFBKMT
ETV1
EXD2
EXO1
EXOC7
EXOSC1
EZH2
FAAP20
FAHD1
FAM102B
FAM110B
FAM124A
FAM135B
FAM151A
FAM153A
FAM169B
FAM20C

FAM213A
FAM224A///FAM224B
FAM3D
FAM47C
FAM63A
FAM71B
FAM71E2
FAM72A///FAM72D///FAM72B///FAM72C
FAM83A
FANCA
FANCC
FANCI
FANCL
FANCM
FARS2
FBF1
FBLIM1
FBXL16
FBXO5
FCAR
FCGBP
FCGR2C
FCHSD2
FEN1
FEV
FGF13-AS1
FGFR2
FKBP8
FLG-AS1
FLI1
FLJ23867///QSOX1
FLJ31104
FLJ32154
FLJ32255
FLJ32742///DCAF8L2
FLJ45513///TAC4
FLOT1
FMN1
FMNL3
FMO2
FNDC5
FNIP2
FOSL2
FOXD1
FOXM1
FOXN3
FOXRED2
FRMD4B
FRMD7
FUT3
FUT6
FUT9
FXR1
FXYD2
G2E3
GAB3

GABPB1
GABRG3-AS1
GAD2
GALNT16
GALNT5
GALR3
GAPDHS
GAREM2
GAS2L3
GATA4
GATAD2A
GATSL3
GDF3
GDF9
GDNF
GFI1B
GGA2
GGT2///GGTLC1///GGTLC2///GGT1
GGT6
GH1///CSHL1///CSH2///CSH1
GH2
GIF
GIPC1
GJC1
GLG1
GLRA3
GLYCTK-AS1
GMNC
GMPS
GNG8
GNL3L
GNRHR
GOLIM4
GOLT1B
GP2
GPLD1
GPR1
GPR12
GPR162
GPR176
GPR19
GPR25
GPR27
GPR62
GREB1
GREB1L
GREM1
GRIN3B
GS1-24F4.2
GSTZ1
GSX1
GTF2IRD2
GTPBP1
GYPB
H1FNT
H2AFX

HAP1
HAUS5
HAUS6
HBBP1
HDAC7
HECW2
HELLS
HENMT1
HEPH
HEPN1///HEPACAM
HERC2P7
HHLA1
HINT3
HIPK2
HIST1H2AH///HIST1H2AG///HIST1H2AM///HIST1H2AL///HIST1H2AK///HIST1H2AI
HIST1H2AK
HIST1H3F///HIST1H3B///HIST1H3H///HIST1H3J///HIST1H3G///HIST1H3I///HIST1H3E///HIST1H3C///HIST1H3D///HIST
HLTF
HM13
HMGA1
HMGB2
HMGN5
HMP19
HNMT
HOXA-AS3
HOXC5
HOXD11
HPGD
HPS6
HPSE2
HR
HSD3B7
HSDL2
HSP90AA1
HTR1F
HYPM
IBA57
IFI30///PIK3R2
IGKC
IGLL1
IGSF11-AS1
IGSF22
IKZF2
IL12RB1
IL16
IL27RA
ING5
INHBA
INPP5D
INPP5J
INPP5K
INSIG1
INSRR
IPO9
IQCH
IRGQ

ISPD-AS1
ISY1
ITGAX
ITGB1
ITGB3
ITPKB
ITPR2
JAK3
JAM3
KALRN
KANK2
KANSL3
KAZN
KCNA4
KCNAB1
KCNIP2
KCNJ16
KCNK7
KCNK9
KCNMA1
KIAA0391
KIAA0825
KIAA1524
KIAA1654
KIAA1671
KIAA2026
KIF13A
KIF14
KIF1C
KIF23
KIF25
KIF4A
KIF5A
KIR2DL3
KIR3DL2///KIR3DL1
KLB
KLF11
KLF15
KLF8
KLF9
KLHL22
KLK11
KLRD1
KMT2A
KMT2D
KNL1
KNTC1
KPNA1
KRT32
KRTAP4-3
KRTAP5-2
KRTAP9-8
LAGE3
LAMA3
LAMB4
LARGE-AS1

LARS2
LCE1E
LCN12
LCN15
LCN8
LDHAL6A
LETM1
LEXM
LGALS12
LHFPL3
LHPP
LHX2
LILRB1
LILRP2
LIN9
LINC00028
LINC00032
LINC00165
LINC00305
LINC00317
LINC00330
LINC00460
LINC00470
LINC00574
LINC00588
LINC00623///LINC00869
LINC00687
LINC00689
LINC00838
LINC00884
LINC00894
LINC00928
LINC01004
LINC01300
LINC01355
LINC01419
LINC01426
LINC01428
LINC01500
LINC01514
LINC01516
LINC01526
LINC01532
LINC01607
LINC01620
LMCD1
LMF1
LOC100127886
LOC100129476
LOC100130741
LOC100130950
LOC100131303
LOC100132078
LOC100133920
LOC100287290
LOC100287590

LOC100288490
LOC100288637///ARHGAP11B
LOC100420758
LOC100421171
LOC100505716
LOC100505774
LOC100505874///MLLT10P1
LOC100506113
LOC100507140
LOC100507194
LOC100507461
LOC100507487
LOC100652931///RBMY2FP
LOC100996624
LOC101926963
LOC101927044
LOC101927123
LOC101927166
LOC101927410
LOC101927516
LOC101927550
LOC101927604
LOC101927620
LOC101927735
LOC101927752
LOC101927876
LOC101928069
LOC101928135
LOC101928185
LOC101928269///LOC100506403///RUNX1
LOC101928417
LOC101928615///FNDC3B
LOC101928647
LOC101928718///FAM212B
LOC101928748
LOC101928844
LOC101928847
LOC101928851
LOC101928910///FAM66C
LOC101928961
LOC101929054
LOC101929162
LOC101929219///LOC100505650///C1orf186
LOC101929372
LOC101929540///LOC101928670///LOC101928344///LOC100996442///LOC100288069///LOC100134822
LOC101929964///LINC01184
LOC101930075///PKD1
LOC101930349///LOC101930344///CGNL1
LOC102723380
LOC102723620
LOC102724362
LOC102724532///SP2-AS1
LOC102800447///LOC101930566
LOC105369739
LOC105369974
LOC105370612

LOC105371708
LOC105373738
LOC105374428
LOC105374768
LOC105376944
LOC145783///ZNF280D
LOC162137
LOC283177
LOC283194
LOC283435
LOC283745
LOC283788
LOC283887
LOC285627
LOC339260
LOC339975
LOC340074
LOC400548
LOC400756
LOC401176
LOC439933
LOC440028
LOC440792
LOC440862///ATP6V1E2
LOC541472
LOC553103
LOC642852
LOC643355
LOC646241
LOC646383
LOC646484
LOC647070
LOC728040
LOC728743
LOC729461///FAM230B///FAM230C
LOC729506
LOC729966///PDE4C
LOC81691
LPIN3
LPP
LRCH3
LRP5
LRP6
LRR1
LRRC4
LRRIQ1
LSG1
LSMEM1
LTBR
LZTS1
MADCAM1
MAGI1
MAGIX
MAL
MALL
MAML3

MAN1A2
MAP1LC3A
MAP3K10
MAPK10
MAPK3
MAPKAPK2
MAPKAPK3
MAPT
MASTL
MBD3L1
MBP
MCF2L
MCHR2
MCM10
MCM2
MCM4
MCM6
MCM8
ME1
MED31
MELK
MERTK
MESP2
MFAP3
MGAM2
MGC16275
MGLL
MIR1204///PVT1
MIR155///MIR155HG
MIR4680///PDCD4
MIR548XHG
MIR6734///ELOVL1
MIR6890///QARS
MIR99AHG
MKI67
MKS1
MLF1
MLLT10
MMEL1
MMP20
MMP24
MMP28
MPHOSPH9
MPI
MPP4
MPST
MRAP
MRAS
MRPL44
MRPL47
MRPS12
MRPS31
MS4A7
MSH6
MSI2
MSL2

MSN
MSR1
MST1L
MTF2
MTFR2
MTHFD2
MTMR3
MTSS1
MTUS2
MUC12
MUC7
MVB12A
MYBL2
MYH11
MYL5
MYNN
MYO16
MYO7B
MYZAP
NAB1
NADK2-AS1
NAPSA
NAV2-AS5
NCAPG2
NCAPH
NCDN
NCR2
NCS1
NDE1
NDRG4
NDST2
NDST3
NDUFA11
NDUFS1
NDUFS7
NEDD4
NEMP1
NENF
NFATC1
NFRKB
NHLRC3
NKX6-1
NLGN3
NLRP1
NLRP14
NLRX1
NMNAT2
NOBOX
NOL3
NOS1
NOVA2
NR4A1
NR6A1
NRAP
NRAV
NRDE2

NRIP3
NSG1
NT5C2
NUDT18
NUF2
NUP133
NUP155
NUP210
NUP50
NUP62
NUTM1
NUTM2B
NXF3
NXNL2
NXPH2
OBP2A
OCM2
ODF3L2
OLAH
OPN4
OPN5
OPRL1
OR10H3
OR13C4
OR1C1
OR2F1
OR2H4P
OR5P3
ORAI2
ORM2///ORM1
OTOF
OTUB2
OVCA2///DPH1
P2RY2
PABPC1L2B///PABPC1L2A
PAFAH1B2
PALLD
PANK2
PAOX
PAPOLB
PARD6G-AS1
PARP2
PARPBP
PCBP1-AS1
PCBP3
PCDHGA10
PDE4A
PDLIM4
PDX1
PEBP1
PEG10
PELO
PEX11G
PEX5
PGPEP1
PHACTR2-AS1

PHF23
PHLDA1
PHYHIP
PIAS2
PIK3C2A
PIK3CA
PIKFYVE
PINK1
PIP5K1A
PITX3
PKMYT1
PLA2G1B
PLA2G4F
PLA2G6
PLA2G7
PLCB1
PLCE1-AS2
PLD4
PLK4
PLOD2
PLSCR1
PLXDC1
PLXNC1
PMCHL2
PNLIPRP1
POLA1
POLE2
POLQ
POLR1D
POLRMT
POU5F1B
POU5F1P4///POU5F1P3///POU5F1B///POU5F1
PPFIBP1
PPIL2
PPL
PPY
PRAP1
PRC1
PRDX2
PRH1-PRR4///PRR4///PRH1
PRIM2
PRO2958
PROZ
PRPS1
PRSS2
PRSS27
PSG11///PSG3
PSIP1
PSMD5
PSMD5-AS1
PTCRA
PTGDR2
PTGES3L
PTGIS
PTK2B
PTPN7

PTPRN2
PVALB
PXDC1
PXT1
PYHIN1
R3HDM1
RAB11B-AS1
RAB11FIP1
RAB21
RAB30
RAB3C
RAB3D
RAD21
RAD51
RAD51AP1
RAD54L
RAI1
RANBP10
RARG
RASAL2
RBL1
RBM15
RBM20
RBM8A
RBMS3
RBP4
RBPJL
RBPMS-AS1
RCBTB1
RECQL
RETNLB
RFC4
RFTN1
RGAG4
RGCC
RGS12
RGS14
RGS7
RHEBL1
RHOD
RHOXF1
RIBC1
RIC3
RIDA
RILPL1///SNRNP35
RIMS1
RMI1
RMI2
RNASEH2B
RNF141
RNF148
RNF167
RNF17
RNF38
RNH1
RNPEPL1

ROGDI
ROR1
RPS15
RPS23
RSRC1
RUNX1T1
S1PR3
SAA3P
SAMD11
SAMD5
SART3
SBF2-AS1
SCAND2P
SCN1B
SCN2B
SCNN1B
SCNN1G
SCPEP1
SCRN2
SCRT1
SEL1L2
SEMA7A
SENP5
SEPT5-GP1BB///SEPT5///GP1BB
SERPINB1
SERPIND1
SFT2D3
SFTA2
SGSM1
SH2D1A
SH3GLB2
SH3PXD2A-AS1
SH3PXD2B
SH3RF2
SHANK2
SIAH1
SIRPA
SKAP2
SLAMF8
SLC11A1
SLC12A4
SLC15A1
SLC16A6
SLC16A9
SLC17A9
SLC22A14
SLC22A24
SLC22A6
SLC22A9
SLC24A1
SLC28A1
SLC30A3
SLC35C1
SLC35C2
SLC35G3
SLC36A4

SLC37A4
SLC38A10
SLC39A11
SLC39A3
SLC39A5
SLC44A4
SLC45A2
SLC46A2
SLC48A1
SLC5A1
SLC6A18
SLC6A2
SLC6A9
SLC7A6
SLC7A7
SLC7A8
SLC9A7
SLF1
SLIT1
SLURP1
SMAD3
SMAGP
SMC1A
SMC4
SMIM1
SMIM5
SMOX
SMPD2
SNHG22
SNORA37
SNORA41///EEF1B2
SNORA68
SNORD24///SNORD36A///SNORD36B///RPL7A
SNORD68///RPL13
SNRNP48
SNRPG
SNX10
SNX25
SNX32
SORBS1
SOSTDC1
SOX1
SOX9-AS1
SPAG5
SPATA13
SPEF2
SPIN2A
SPINK5
SPTBN1
SPTLC3
SRRM3
SRSF1
ST18
ST7-OT4
ST8SIA5
STAT1

STAU2-AS1
STGC3
STK24
STK3
STK32B
STK35
STK36
STPG2
SUFU
SULT2B1
SULT4A1
SUMF1
SUZ12
SVOPL
SYCP2
SYNGR1
SYNGR3
SYNJ2
SYNRG
SYT11
SYT6
SZRD1
TAAR2
TAAR5
TACR1
TAF15
TAF2
TAF3
TAGLN3
TAL2
TANGO6
TARDBP
TBC1D15
TBC1D31
TBK1
TBX20
TBX3
TCAM1P
TCEANC
TCF3
TCF7
TCL6
TEF
TEN1-CDK3///TEN1
TES
TEX28
TFDP2
TGIF1
THBS1
THRA
THSD4
TICRR
TIMELESS
TLL2
TLR3
TM4SF5

TM6SF2
TM7SF2
TMBIM4
TMCO2
TMCO3
TMED5
TMEM107
TMEM108
TMEM132C
TMEM150A
TMEM165
TMEM184C
TMEM229B
TMEM253
TMEM39A
TMEM61
TMEM62
TMEM74
TMPRSS11B
TMPRSS2
TNFAIP6
TNFRSF8
TNFSF13B
TNNC2
TNRC6B
TNXB
TOLLIP
TOM1
TONSL
TOP2A
TOP3B
TOPBP1
TP53I11
TP53I3
TPH2
TPRG1
TPRXL
TPSAB1
TPT1P8
TPTE
TRAF1
TRIM14
TRIP13
TRPM1
TRPT1
TSEN54
TSPAN3
TSPAN31
TSPY1
TSPY10///TSPY8///TSPY4///TSPY3///TSPY1
TSR3
TSSK2///DGCR14
TST
TTC22
TTC23
TTC26

TTC39A
TTLL11
TTTY11
TTTY7
TVP23C-CDRT4///CDRT4///TVP23C
TXNRD1
TYMS
U2SURP
UBA2
UBA6
UBE2F
UBE2I
UBL3
UBOX5
UGGT1
UGT1A3///UGT1A1///UGT1A9///UGT1A5///UGT1A8
UGT2A2///UGT2A1
UGT3A1
UHRF1
UHRF1BP1
UMPS
UNC45A
UNC5CL
UPK1A
USF3
USP1
USP18
USP5
UTS2B
VDR
VHL
VN1R1
VPS13B
VPS18
VPS26B
VSIG2
VSTM4
WBP11
WDHD1
WDR5B
WDR7
WFDC8
WHSC1
WNT16
WNT6
WSCD2
WTAP
WWC2-AS2
YES1
YIPF2
YME1L1
ZBED2
ZBTB10
ZBTB49
ZC3H3
ZC4H2

ZCCHC2
ZCWPW1
ZDHHC3
ZFAT
ZFR2
ZFYVE16
ZG16B
ZIC2
ZIC4
ZMAT5
ZMYM6
ZNF160
ZNF185
ZNF224
ZNF233
ZNF264
ZNF284
ZNF300P1
ZNF362
ZNF367
ZNF391
ZNF439
ZNF441
ZNF496
ZNF506
ZNF507
ZNF551
ZNF552
ZNF561///ZNF562
ZNF597
ZNF626
ZNF653
ZNF667
ZNF672
ZNF677
ZNF70
ZNF709
ZNF710
ZNF775
ZNF843
ZNF844
ZNF91
ZNF99
ZP4
ZSCAN12
ZSCAN2
ZSCAN32
ZWILCH
ZYG11A
