# Supplementary material for: Application of Protein-Protein Interaction Network Analysis in Order to Identify Cervical Cancer miRNA and mRNA Biomarkers
Source: ScientificWorldJournal. 2023 Sep 14;2023:6626279. doi: 10.1155/2023/6626279 (PMC10513823; doi:10.1155/2023/6626279)
Supplement: Supplementary Materials — This paper includes four supplementary files named S1 to S4. [file 6626279.f1.zip › Supplementary file S2 (1).pdf]

Cervical cancer driver genes

A1CF
A2ML1
AATF
ABCA13
ABCA3
ABCA5
ABCB1
ABCC1
ABCC10
ABCC5
ABCC6
ABCD1
ABCF1
ABCF3
ABCG1
ABCG8
ABHD8
ABI2
ABL1
ABL2
ABR
ACACA
ACAD10
ACAN
ACLY
ACO1
ACOX1
ACSL3
ACTB
ACTG1
ACTN2
ACTN3
ACTN4
ACTRT1
ADAM29
ADAM32
ADAMTS1
ADAMTS10
ADAMTS12
ADAMTS16
ADAMTS17
ADAMTS18
ADAMTS3
ADAMTS6
ADAMTS7
ADAMTS9
ADAMTSL1

ADAMTSL2
ADAMTSL3
ADARB1
ADD1
ADGRB1
ADGRF1
ADGRG2
ADGRG7
ADGRV1
ADSSL1
AEBP1
AEBP2
AFF1
AFF4
AGAP2
AGAP3
AGAP4
AGBL5
AGER
AGO2
AGTPBP1
AHCYL1
AHNAK
AHNAK2
AIFM1
AJUBA
AK7
AKAP13
AKT1
ALAS1
ALAS2
ALDH1A3
ALDOA
ALG13
ALG1L
ALG6
ALMS1
ALS2
ALX1
AMPH
ANAPC2
ANGPT4
ANK2
ANK3
ANKFY1
ANKRD12
ANKRD13D
ANKRD30A
ANKRD36

ANKRD6
ANO3
ANO4
ANO8
ANOS1
ANXA2
ANXA4
AOAH
AP002748.4
AP1M1
AP2A1
AP3B1
AP3D1
APAF1
APBA2
APBB1
APBB1IP
APBB2
APC
APOB
APOBEC3C
APOBEC3G
APOH
ARAP1
ARFGEF1
ARGFX
ARHGAP29
ARHGAP30
ARHGAP4
ARHGAP5
ARHGEF10L
ARHGEF17
ARHGEF18
ARHGEF2
ARHGEF5
ARID1A
ARID1B
ARID2
ARIH2
ARMC5
ARMC9
ARMCX4
ASB18
ASMTL
ASPM
ASPN
ASXL1
ATAD2B
ATG2A

ATL3
ATM
ATMIN
ATP10A
ATP1A3
ATP1B3
ATP2A1
ATP2B2
ATP6V0A4
ATP6V1A
ATP7B
ATP8B2
ATP8B3
ATRAID
ATRX
ATXN1L
ATXN2L
AURKA
AXIN2
B2M
B3GNT5
B3GNT8
BAG4
BAG5
BAG6
BAHD1
BAIAP2L1
BAP1
BAZ1B
BAZ2A
BCAM
BCL3
BCL6
BCL9
BICC1
BLM
BLOC1S6
BMP8B
BNC2
BNIP3
BNIPL
BOD1L1
BRAP
BRCA2
BRDT
BRPF1
BRPF3
BRS3
BRWD1

BRWD3
BSDC1
BTBD1
BTK
BYSL
C12orf41
C12orf43
C1GALT1C1
C20orf27
C2CD6
C2orf16
C2orf78
C3orf70
C6
CA11
CA7
CACNA1A
CACNA1B
CACNA1C
CACNA1I
CACNA2D1
CACNA2D3
CAD
CALR
CAMLG
CAPSL
CARD11
CARMIL2
CASC3
CASP8
CAVIN3
CBL
CBLC
CBWD5
CC2D1A
CCAR1
CCAR2
CCDC105
CCDC114
CCDC120
CCDC134
CCDC144A
CCDC148
CCDC168
CCDC180
CCDC86
CCDC87
CCND1
CCSER2

CCT5
CCT6B
CD101
CD244
CD3EAP
CD63
CD74
CDC123
CDC25B
CDC37
CDC42BPG
CDC42EP4
CDCA2
CDH12
CDH19
CDH23
CDH9
CDHR3
CDK12
CDK13
CDK14
CDK16
CDK5RAP2
CDKAL1
CDKL5
CDKN1A
CDT1
CELF3
CELSR1
CENPB
CENPF
CEP170
CEP19
CEP295
CEP295NL
CEP350
CFAP54
CFAP57
CFAP61
CFAP65
CFH
CFHR5
CFI
CFP
CGAS
CHAD
CHD1
CHD5
CHEK2

CHN2
CHRM1
CHRM2
CHRNA5
CHUK
CIC
CIZ1
CKAP2
CLASP1
CLCN2
CLEC7A
CLK3
CLMN
CLPB
CLSPN
CLTC
CLTCL1
CMKLR1
CNBD2
CNGA2
CNNM4
CNOT1
CNOT3
CNTLN
CNTN1
CNTN3
CNTN4
CNTNAP2
CNTNAP3
CNTNAP5
CNTRL
COASY
COG5
COL11A1
COL11A2
COL12A1
COL18A1
COL19A1
COL1A1
COL1A2
COL20A1
COL24A1
COL25A1
COL27A1
COL3A1
COL4A3
COL4A3BP
COL6A1
COL6A2

COL7A1
COL8A1
COMMD3-BMI1
COQ4
CORIN
CORO1A
CORO1B
CPEB4
CPED1
CPN1
CPNE4
CPSF1
CPT1A
CPXM1
CPZ
CR1
CRB1
CRB2
CREBBP
CRTC1
CRTC3
CS
CSE1L
CSF1R
CSMD2
CSMD3
CSNK2B
CSTF2
CTAGE6
CTC1
CTCF
CTDSPL2
CTNNA1
CTNNA2
CTNNA3
CTNNB1
CTNND1
CTR9
CUBN
CUL1
CUL2
CUL4B
CXCL13
CXorf67
CXXC1
CYFIP2
CYP2B6
CYP2U1
DAAM1

DACH2
DALRD3
DAZAP1
DBF4B
DCAF12L1
DCAF6
DCC
DCHS1
DCHS2
DCP1B
DCT
DDR1
DDX21
DDX3X
DENND5A
DES
DGCR8
DGKD
DGKI
DGKQ
DHPS
DHRSX
DHTKD1
DHX16
DHX29
DHX57
DHX8
DHX9
DIAPH1
DIAPH3
DIDO1
DIP2B
DISP3
DLC1
DLG4
DLGAP2
DLGAP3
DLGAP5
DMBT1
DMD
DNAAF2
DNAH1
DNAH11
DNAH14
DNAH2
DNAH3
DNAH7
DNAI2
DNAJA2

DNAJB12
DNAJC1
DNAJC14
DNM2
DOCK11
DOCK2
DOK3
DOLK
DPEP2
DPP4
DPP9
DPYSL2
DRP2
DSC2
DSCAM
DSCAML1
DSE
DSP
DST
DUS3L
DUSP6
DVL1
DYM
DYNC1H1
DYNC1I1
DYSF
E2F3
EBF4
ECD
ECE2
ECHDC2
ECT2L
EDARADD
EDC3
EDRF1
EEF1A1
EEF2K
EFCAB5
EGR1
EHHADH
EHMT2
EIF2AK1
EIF3E
EIF4A1
EIF4ENIF1
EIF5
EIF5B
ELAVL4
ELFN2

ELMSAN1
ELOA
ELOA2
EMILIN2
EML6
ENAH
ENO1
ENO3
ENPEP
EP300
EPAS1
EPB41L1
EPB41L2
EPB41L4B
EPHA10
EPHB1
EPHB2
EPHB3
EPHB4
EPHB6
EPN1
EPPK1
EPRS
EPS15
EPS15L1
ERBB2
ERBB3
ERBB4
ERBIN
ERCC3
ERICH6
ERN1
ESPL1
ESYT3
EVPL
EXOC1
EXOC3-AS1
EXOC3L2
EXOC5
EXPH5
F2RL3
F8
FAAP100
FAM120A
FAM120B
FAM129A
FAM13A
FAM170B
FAM171A2

FAM208A
FAM214A
FAM234B
FAM47A
FAM71B
FAM83G
FAM98A
FAM9A
FANCB
FANCL
FANCM
FAS
FASN
FAT1
FAT2
FAT3
FBF1
FBN1
FBN2
FBN3
FBXL20
FBXO11
FBXO41
FBXO42
FBXW7
FCGBP
FCHO2
FCHSD1
FGA
FGFR2
FKBP15
FLG
FLII
FLNA
FLNC
FLOT1
FLT3
FN1
FNBP4
FOXA2
FOXK1
FOXO4
FRAS1
FREM1
FREM2
FRMD6
FRMPD3
FUBP1
FZD2

GABBR2
GABRG1
GAD1
GAK
GALC
GALNT13
GCM2
GCN1
GCNA
GCNT2
GDI2
GFRA3
GGT5
GGT7
GHDC
GHR
GIGYF1
GIPR
GJA9
GLRX5
GLT1D1
GLYR1
GMEB1
GMIP
GNA15
GNAI3
GNAQ
GNAS
GNPTAB
GNS
GOLGA6L10
GOLGA6L22
GOLGA6L6
GOLGB1
GON4L
GPAA1
GPCPD1
GPHN
GPKOW
GPR139
GPR158
GPR176
GPR39
GPR87
GPRASP1
GPRASP2
GPRIN1
GPS1
GRAMD2A

GRAMD2B
GREB1L
GRIA4
GRID2
GRIK3
GRIK5
GRIN2A
GRIN2B
GRIN2C
GRK7
GRM5
GRM7
GSK3A
GSN
GSPT1
GSR
GSTP1
GTDC1
GTF2E1
GTF2IRD1
GTF2IRD2B
GTF3C1
GTPBP6
GUCY1A3
H2BFWT
HAP1
HAUS6
HCN2
HDLBP
HDX
HEATR1
HEATR4
HECTD3
HECTD4
HECW2
HELQ
HELZ
HERC2
HERC6
HEXIM1
HGF
HGS
HIBCH
HIF3A
HIST1H2AL
HIST2H2AB
HIVEP1
HLA-A
HLA-B

HLA-C
HLA-DRA
HLA-DRB5
HMCN1
HMCN2
HMGA1
HMGXB4
HNRNPA1
HNRNPM
HOXA4
HP1BP3
HPD
HPS5
HRASLS
HRC
HRH1
HRNR
HSD17B4
HSP90AA1
HSP90AB1
HSP90B1
HSPA12B
HSPA1B
HSPA4
HSPA4L
HSPA8
HSPB1
HSPG2
HSPH1
HTATSF1
HTR6
HTT
HUWE1
IARS2
ICAM5
IDS
IFNAR2
IFNGR1
IFNL2
IGF1R
IGF2BP2
IGF2BP3
IGFALS
IGSF10
IGSF9B
IKBKE
IL1A
IL1RAPL2
IL20RB

IL23R
IL4R
INPP5D
INPPL1
INSR
INTS6L
INTS7
INVS
IPO5
IPO7
IPO9
IQCE
IQCG
IQGAP2
IQGAP3
IQSEC2
IQSEC3
IRF1
IRF2BPL
IRS1
ISL1
ITCH
ITGA11
ITGA8
ITGAE
ITGAL
ITGAV
ITGAX
ITGB4
ITGB5
ITIH1
ITIH5
ITPR3
JAG1
JAG2
JAK2
JAKMIP1
JMJD1C
JPH2
JUND
JUP
KALRN
KAT2A
KCNA1
KCNA4
KCNC3
KCND1
KCNH2
KCNH4

KCNH5
KCNQ2
KDM3B
KDM4C
KDM5B
KDM5C
KDM6A
KDR
KEAP1
KHDC4
KIAA0825
KIAA1210
KIAA1217
KIAA1522
KIAA1549L
KIAA1551
KIAA1671
KIAA1683
KIF11
KIF14
KIF17
KIF1A
KIF1B
KIF5C
KIF7
KIT
KLC2
KLF5
KLHDC10
KLHDC7A
KLHL29
KLHL36
KLHL4
KLHL6
KMT2B
KMT2C
KMT2D
KMT2E
KNTC1
KPNA1
KPNA6
KRAS
KRI1
KRT1
KRT14
KRT28
KRT6B
KRT7
KRT83

KRT84
KRTAP9-4
KTI12
L1CAM
L1TD1
LAMA2
LAMA3
LAMA5
LAMB2
LAMC1
LAMC2
LAMC3
LAS1L
LATS2
LDB1
LGALS8
LGR5
LIG1
LIMD1
LIN9
LINGO1
LINS1
LIPH
LMX1A
LONRF1
LPA
LRBA
LRFN1
LRFN5
LRIT1
LRP1
LRP1B
LRP2
LRP4
LRP5
LRP6
LRRC55
LRRC9
LRRIQ3
LRRK1
LRRN2
LTBP3
LYN
MACF1
MADD
MAG
MAGEA3
MAGEB10
MAGEB3

MAGEC3
MAGEE1
MAGEF1
MAGEL2
MAML1
MAML2
MAN1B1
MAP1A
MAP1B
MAP3K1
MAP3K11
MAP3K13
MAP3K15
MAP3K3
MAP3K6
MAP7D3
MAP9
MAPK1
MAPK15
MAPKBP1
MARCH6
MARCKS
MARK1
MARK2
MAST1
MATR3
MB21D2
MBD1
MBD4
MBD5
MBNL1
MCCC1
MCM10
MCM2
MDC1
MDH1
MED1
MED12
MED12L
MED13
MED26
MEF2D
MEGF10
MEGF8
MELK
MEOX2
MEP1A
MET
METAP1

MEX3D
MFAP1
MFSD8
MGAM2
MGAT3
MGEA5
MGMT
MIA3
MICAL3
MICB
MINK1
MIS18BP1
MKI67
MKX
MLC1
MLF1
MLNR
MME
MMEL1
MMP11
MMP2
MNAT1
MOB3C
MORC1
MPDZ
MPEG1
MPHOSPH10
MPND
MPP7
MPZL1
MRC1
MRLN
MRPL22
MRPS12
MS4A5
MSH4
MST1
MST1R
MT1H
MTDH
MTHFR
MTMR1
MTMR10
MTMR3
MTMR7
MTOR
MUC1
MUC12
MUC16

MUC3A
MUC4
MUC5AC
MUC5B
MUSK
MVB12B
MX1
MXRA5
MYBBP1A
MYBL2
MYBPC2
MYH13
MYH14
MYH2
MYH3
MYH4
MYH9
MYLK
MYO15A
MYO15B
MYO18A
MYO5B
MYO7A
MYO7B
MYO9B
MYOCD
MYOF
MYOM2
MYOM3
N4BP2
NAALAD2
NAALADL2
NACAD
NADK
NADSYN1
NALCN
NAT10
NAV1
NAV2
NBEAL2
NBPF1
NBPF10
NBPF14
NBPF26
NBPF9
NCK1
NCL
NCOA1
NCOA2

NCOA5
NCOR1
NCOR2
NDC80
NDST3
NDST4
NEB
NECTIN2
NEDD4L
NEK1
NEK8
NELFA
NELFE
NES
NF1
NFE2L2
NFE2L3
NFX1
NHS
NID1
NIM1K
NIPAL1
NIPBL
NKAPL
NLGN1
NLRC3
NLRC5
NLRP5
NLRP9
NLRX1
NME8
NMNAT3
NMT2
NOD1
NOD2
NOS1
NOS3
NOTCH1
NOTCH2
NOTCH3
NPAS2
NPHP4
NPHS1
NPIPB4
NPTX2
NR1H2
NR2C1
NR3C1
NRCAM

NRXN2
NSD1
NT5E
NTRK1
NTRK3
NUMA1
NUP155
NUP210L
NUP88
NUP98
NVL
NWD2
NXF1
OAS3
OBSCN
OCRL
OFD1
OGT
OIT3
OLFM3
OR10G7
OR10S1
OR10W1
OR4Q3
OR52L1
OR56A5
OR56B4
OR5M11
OR5M3
OR7G1
OR8K5
OSBPL3
OSBPL8
OSMR
OTOF
OTUD3
OVCH1
OXR1
P2RX1
P2RY1
PABPC1L
PACS2
PACSIN3
PAK3
PALM2-AKAP2
PALMD
PAPLN
PAPSS1
PAPSS2

PARD3B
PARPBP
PASK
PATL1
PAX1
PAXIP1
PBDC1
PBRM1
PCDH19
PCDH20
PCDHA1
PCDHA12
PCDHA2
PCDHA5
PCDHA6
PCDHA7
PCDHA8
PCDHA9
PCDHAC1
PCDHB13
PCDHB14
PCDHB15
PCDHB2
PCDHB5
PCDHB6
PCDHGA10
PCDHGA2
PCDHGA7
PCDHGA9
PCDHGB2
PCDHGB3
PCDHGB6
PCLO
PCM1
PCNT
PCNX2
PCSK4
PCSK7
PDE6B
PDGFRA
PDGFRB
PDHA2
PDIA4
PDIA6
PDK1
PDLIM1
PDLIM5
PDP1
PDS5B

PDSS1
PDZD7
PDZRN4
PELI3
PELO
PER2
PERM1
PES1
PEX11A
PEX7
PGAP1
PGM2
PGR
PHACTR4
PHC2
PHEX
PHF1
PHF12
PHKA2
PHLDA2
PHRF1
PIDD1
PIEZO1
PIGK
PIGN
PIGX
PIK3C2A
PIK3C2B
PIK3CA
PIK3R1
PIK3R4
PINK1
PIP5K1C
PKD1L1
PKDREJ
PKHD1
PKHD1L1
PKN2
PKNOX1
PKP3
PLAA
PLBD1
PLCB3
PLCG1
PLCH1
PLCL1
PLD1
PLD5
PLEC

PLEKHG1
PLEKHG7
PLEKHN1
PLK2
PLXNA1
PLXNB2
PLXNC1
PLXND1
PML
PNISR
PNLDC1
POLA1
POLD3
POLR1B
POM121
POM121C
POM121L12
PON3
POTEB3
POTEH
POU2F1
POU4F1
POU4F2
POU4F3
POU6F1
PPA2
PPEF1
PPIH
PPIL4
PPM1J
PPP1R12A
PPP1R16B
PPP1R1C
PPP1R26
PPP1R3A
PPP2R3A
PPP2R5C
PPP6R1
PRAG1
PRAMEF2
PRDM15
PRDM7
PRDM9
PREP
PREX1
PRKAR2A
PRKCA
PRKCD
PRKCI

PRKCZ
PRKD1
PRKDC
PROM2
PROS1
PRPF38B
PRPF8
PRR14L
PRX
PSEN1
PSG9
PSMD8
PSME3
PSME4
PTBP1
PTEN
PTGES3L-AARSD1
PTK2B
PTPN11
PTPN14
PTPN21
PTPN22
PTPRB
PTPRQ
PTPRT
PUS1
PUS7
PWWP2A
PXDNL
PXMP4
PYGL
PZP
QRICH2
QTRT1
RABGAP1L
RABGEF1
RAD18
RAD21
RAD50
RAD54B
RAD54L2
RAG2
RAI1
RANBP2
RANBP6
RAPGEF2
RAPGEF5
RAPGEFL1
RARG

RARS2
RASIP1
RB1
RB1CC1
RBBP6
RBBP7
RBBP8
RBFOX1
RBM14
RBM33
RBM39
RBP1
RBP3
RBPMS
RECQL5
RELA
REPS2
RESF1
RET
REV3L
RFC1
RFX7
RGPD3
RGPD4
RGPD8
RGS3
RHD
RICTOR
RIMBP2
RIMKLB
RMI1
RNF139
RNF168
RNF213
RNF219
RNF40
RNH1
ROBO1
ROBO3
ROCK2
ROR2
RPGRIP1
RPL39L
RPL7
RPLP0
RPRD2
RPS16
RPS7
RRAD

RREB1
RRP12
RS1
RSC1A1
RSL1D1
RSRC1
RTF1
RUNX1T1
RUSC1
RYR1
SAFB
SALL3
SALL4
SAMD4A
SAMD9
SASH3
SASS6
SBDS
SBF2
SCAF4
SCAMP3
SCLT1
SCN7A
SCRIB
SCYL2
SDAD1
SDC1
SDK1
SEC16A
SEC23IP
SEC24D
SEMA4G
SEMA5A
SEMA5B
SENP2
SENP6
SERINC3
SERPINA3
SERPINF1
SERPING1
SETD2
SETX
SEZ6L
SF1
SF3B1
SFN
SGO2
SGSM3
SH3GLB1

SH3KBP1
SH3PXD2B
SHANK1
SHC2
SHKBP1
SHROOM3
SI
SIGLEC10
SIGLEC7
SIGLEC8
SIN3A
SIPA1L3
SKI
SKIL
SKIV2L
SKOR2
SLAIN2
SLAMF1
SLC12A2
SLC16A13
SLC17A8
SLC18A3
SLC22A11
SLC26A3
SLC26A6
SLC34A1
SLC38A11
SLC44A2
SLC44A4
SLC4A7
SLC52A3
SLC5A11
SLC5A2
SLC6A17
SLC6A19
SLC6A2
SLC9A1
SLC9A7
SLCO2A1
SLCO6A1
SLFN11
SLIT1
SLIT2
SLIT3
SLITRK1
SLITRK2
SLITRK3
SMAD4
SMAD5

SMARCC1
SMC1A
SMC1B
SMG1
SMG5
SMG6
SMS
SNAPC4
SND1
SNRPA
SNX6
SOCS6
SORBS1
SORBS2
SORL1
SOS1
SOX11
SOX2
SOX3
SOX9
SP1
SPAG1
SPAG17
SPAG5
SPAG9
SPATA16
SPATA31A1
SPDYE2
SPEG
SPEN
SPG7
SPHK2
SPINK5
SPINT2
SPON1
SPSB2
SPSB4
SPTB
SPTBN4
SPTBN5
SPTLC2
SRC
SRCIN1
SREBF1
SRP68
SRP72
SRPX2
SRRM1
SRRM2

SRRT
SRSF1
SRSF2
SRSF6
SSPO
SSR3
STAB1
STAG1
STAG2
STARD8
STARD9
STIL
STK10
STK11
STK24
SUPT5H
SVEP1
SVIL
SYCP1
SYDE2
SYNCRIP
SYNE1
SYNE2
SYNE4
SYNPO
SYNPO2L
SYT10
SYT3
SZT2
T
TACC1
TADA2B
TAF1
TAF1B
TAPBP
TARBP1
TARDBP
TARS
TARSL2
TBC1D10B
TBC1D31
TBC1D4
TBC1D8
TBC1D9B
TBX2
TCAP
TCERG1
TCF12
TCF20

TCOF1
TCTEX1D2
TCTN2
TECPR1
TENM3
TERF1
TERT
TEX10
TEX11
TEX14
TEX15
TFRC
TG
TGFBR2
TGM3
TGM4
THAP7
THSD1
TIE1
TIMP1
TJAP1
TJP2
TJP3
TKT
TLE4
TLN1
TMC6
TMC8
TMCO3
TMED9
TMEM131
TMEM132C
TMEM185A
TMEM189
TMEM209
TMEM260
TMEM43
TMEM44
TMEM47
TMEM63C
TMPRSS7
TMPRSS9
TMTC3
TMTC4
TNC
TNFRSF1A
TNFRSF21
TNFSF10
TNIK

TNK2
TNKS1BP1
TNN
TNRC6B
TNS3
TNS4
TNXB
TONSL
TOP2A
TOP3B
TOPBP1
TOR1A
TOX
TP53
TP53BP1
TP63
TP73
TPCN1
TPR
TRA2A
TRAF2
TRAF3
TRAF3IP2
TRAP1
TRERF1
TRIM28
TRIM58
TRIM67
TRIP11
TRIP12
TRIP13
TRMT2A
TRO
TRPM3
TRPM4
TRPM8
TRPS1
TRPV4
TRPV6
TRUB2
TSC2
TSHZ3
TSNARE1
TSPOAP1
TSPYL5
TTC17
TTC21A
TTC39C
TTC7B

TTF1
TTI1
TTK
TTN
TUBB2A
TUFM
TUT7
UBAP2
UBE2O
UBE3A
UBE3C
UBQLN1
UBR2
UBR3
UBR5
UBXN4
UEVLD
UGGT2
UGT2B17
UHRF1BP1L
ULK4
UMODL1
UNC13D
UPF2
URB1
USH1C
USH2A
USP1
USP11
USP28
USP31
USP32
USP33
USP34
USP4
USP42
USP44
USP8
USP9X
UTRN
UVRAG
VARS
VAV2
VAV3
VEGFC
VILL
VLDLR
VN1R1
VPS13B

VPS52
VRTN
VSNL1
VWF
WAC
WASF3
WDFY4
WDPCP
WDR20
WDR33
WDR47
WDR55
WDR61
WDR63
WDR7
WDR72
WDR81
WDTC1
WNK2
WRAP73
WSCD1
XDH
XIAP
XIRP1
XPNPEP1
XPO4
XPO6
XPO7
XRCC3
XRN1
XRN2
YARS
YEATS2
YIPF3
YME1L1
YTHDC1
YWHAE
ZBBX
ZBED4
ZBED9
ZBTB21
ZBTB49
ZC3H11A
ZC3H12B
ZC3H12C
ZC3H18
ZC3H3
ZC3HAV1
ZCCHC8

ZEB2
ZFHX3
ZFP14
ZFP62
ZFP91
ZFP92
ZHX3
ZIC3
ZNF12
ZNF160
ZNF189
ZNF214
ZNF229
ZNF236
ZNF268
ZNF287
ZNF318
ZNF329
ZNF334
ZNF347
ZNF350
ZNF37A
ZNF404
ZNF408
ZNF417
ZNF419
ZNF43
ZNF433
ZNF440
ZNF461
ZNF468
ZNF469
ZNF471
ZNF483
ZNF518B
ZNF519
ZNF536
ZNF541
ZNF546
ZNF548
ZNF550
ZNF551
ZNF559
ZNF564
ZNF565
ZNF566
ZNF567
ZNF568
ZNF569

ZNF573
ZNF578
ZNF597
ZNF600
ZNF608
ZNF615
ZNF627
ZNF645
ZNF658
ZNF675
ZNF676
ZNF681
ZNF689
ZNF69
ZNF714
ZNF728
ZNF750
ZNF773
ZNF780A
ZNF788
ZNF804A
ZNF813
ZNF814
ZNF829
ZNF835
ZNF84
ZNF85
ZNF862
ZNF879
ZNF90
ZNF91
ZNF92
ZNF98
ZNF99
ZNFX1
ZSCAN5A
ZSWIM2
ZSWIM3
ZSWIM4
ZYX
ZZEF1
ZZZ3
AHNAK
CLSPN
CNTNAP2
FAS
MAPK1
MUC4
MYL9

NAV3
PDLIM5
PIK3CA
SLC25A17
TIMM50
