# Supplementary material for: Application of Protein-Protein Interaction Network Analysis in Order to Identify Cervical Cancer miRNA and mRNA Biomarkers
Source: ScientificWorldJournal. 2023 Sep 14;2023:6626279. doi: 10.1155/2023/6626279 (PMC10513823; doi:10.1155/2023/6626279)
Supplement: Supplementary Materials — This paper includes four supplementary files named S1 to S4. [file 6626279.f1.zip › Supplementary file S3 (1).pdf]

miRNA-mRNA interactions

miRNA	Target gene
hsa-miR-1296-5p	MCM2
hsa-miR-31-5p	MCM2
hsa-miR-1-3p	MCM2
hsa-miR-34a-5p	MCM2
hsa-miR-1226-3p	MCM2
hsa-miR-501-3p	MCM2
hsa-miR-500a-5p	MCM2
hsa-miR-615-3p	MCM2
hsa-miR-423-3p	MCM2
hsa-miR-145-5p	MCM2
hsa-miR-193b-3p	MCM4
hsa-miR-193b-3p	MCM4
hsa-miR-1-3p	MCM4
hsa-miR-34a-5p	MCM4
hsa-miR-24-3p	MCM4
hsa-miR-501-3p	MCM4
hsa-miR-31-3p	MCM4
hsa-miR-324-3p	MCM4
hsa-miR-320a	MCM4
hsa-miR-183-5p	MCM4
hsa-let-7b-5p	MCM4
hsa-miR-633	MCM4
hsa-miR-3613-3p	MCM4
hsa-miR-5590-5p	MCM4
hsa-miR-373-5p	MCM4
hsa-miR-616-5p	MCM4
hsa-miR-371b-5p	MCM4
hsa-miR-6822-3p	MCM4
hsa-miR-6502-5p	MCM4
hsa-miR-1301-5p	MCM4
hsa-miR-372-5p	MCM4
hsa-miR-371a-5p	MCM4
hsa-miR-1285-5p	MCM4
hsa-miR-539-5p	MCM4
hsa-miR-1251-5p	MCM4
hsa-miR-517-5p	MCM4
hsa-miR-3675-3p	MCM4
hsa-miR-216b-5p	MCM4
hsa-miR-548c-3p	MCM4
hsa-miR-5571-3p	MCM4
hsa-miR-1206	MCM4
hsa-miR-4284	MCM4
hsa-miR-6829-3p	MCM4
hsa-miR-6791-3p	MCM4
hsa-miR-2276-3p	MCM4

hsa-miR-4638-5p	MCM4
hsa-miR-6741-3p	MCM4
hsa-miR-1281	MCM4
hsa-miR-4485-5p	MCM4
hsa-miR-6890-3p	MCM4
hsa-miR-129-5p	MCM4
hsa-miR-1304-3p	MCM4
hsa-miR-137	MCM4
hsa-miR-3672	MCM4
hsa-miR-4524a-3p	MCM4
hsa-miR-4639-3p	MCM4
hsa-miR-6794-3p	MCM4
hsa-miR-6864-3p	MCM4
hsa-miR-8055	MCM4
hsa-miR-193b-3p	MCM6
hsa-miR-193b-3p	MCM6
hsa-miR-1-3p	MCM6
hsa-miR-215-5p	MCM6
hsa-miR-34a-5p	MCM6
hsa-miR-192-5p	MCM6
hsa-miR-1180-3p	MCM6
hsa-miR-455-3p	CDC45
hsa-miR-575	CDC45
hsa-miR-192-5p	MCM10
hsa-miR-193b-3p	MCM10
hsa-miR-193b-3p	MCM10
hsa-miR-148b-3p	MCM10
hsa-miR-215-5p	MCM10
hsa-miR-24-3p	MCM10
hsa-miR-296-3p	MCM10
hsa-miR-615-3p	MCM10
hsa-miR-34a-5p	MCM10
hsa-miR-185-5p	MCM10
hsa-miR-6510-3p	MCM10
hsa-miR-6888-3p	MCM10
hsa-miR-4522	MCM10
hsa-miR-3120-5p	MCM10
hsa-miR-372-5p	MCM10
hsa-miR-371a-5p	MCM10
hsa-miR-616-5p	MCM10
hsa-miR-373-5p	MCM10
hsa-miR-371b-5p	MCM10
hsa-miR-3613-3p	MCM10
hsa-miR-3664-5p	MCM10
hsa-miR-4432	MCM10
hsa-miR-26b-5p	WDHD1
hsa-miR-218-5p	WDHD1
hsa-miR-193b-3p	MCM8
hsa-miR-155-5p	MCM8

hsa-miR-155-5p	MCM8
hsa-miR-99a-5p	MCM8
hsa-miR-374a-5p	MCM8
hsa-miR-1273e	MCM8
hsa-miR-1273e	MCM8
hsa-miR-6807-5p	MCM8
hsa-miR-6807-5p	MCM8
hsa-miR-4266	MCM8
hsa-miR-4266	MCM8
hsa-miR-6894-5p	MCM8
hsa-miR-6894-5p	MCM8
hsa-miR-4683	MCM8
hsa-miR-4683	MCM8
hsa-miR-1233-3p	MCM8
hsa-miR-1233-3p	MCM8
hsa-miR-1225-3p	MCM8
hsa-miR-1225-3p	MCM8
hsa-miR-6840-3p	MCM8
hsa-miR-6840-3p	MCM8
hsa-miR-665	MCM8
hsa-miR-665	MCM8
hsa-miR-4768-3p	MCM8
hsa-miR-4768-3p	MCM8
hsa-miR-4779	MCM8
hsa-miR-4779	MCM8
hsa-miR-6888-5p	MCM8
hsa-miR-6888-5p	MCM8
hsa-miR-4695-5p	MCM8
hsa-miR-4695-5p	MCM8
hsa-miR-574-5p	MCM8
hsa-miR-574-5p	MCM8
hsa-miR-574-5p	MCM8
hsa-miR-574-5p	MCM8
hsa-miR-943	MCM8
hsa-miR-943	MCM8
hsa-miR-3136-5p	MCM8
hsa-miR-3136-5p	MCM8
hsa-miR-4439	MCM8
hsa-miR-4439	MCM8
hsa-miR-6867-5p	MCM8
hsa-miR-6867-5p	MCM8
hsa-miR-6867-5p	MCM8
hsa-miR-6867-5p	MCM8
hsa-miR-892a	MCM8
hsa-miR-892a	MCM8
hsa-miR-4460	MCM8
hsa-miR-4727-3p	MCM8
hsa-miR-6791-3p	MCM8
hsa-miR-6829-3p	MCM8

hsa-miR-3653-5p	MCM8
hsa-miR-3677-5p	MCM8
hsa-miR-6888-3p	MCM8
hsa-miR-6888-3p	MCM8
hsa-miR-6888-3p	MCM8
hsa-miR-4711-3p	MCM8
hsa-miR-4711-3p	MCM8
hsa-miR-4711-3p	MCM8
hsa-miR-3120-5p	MCM8
hsa-miR-3120-5p	MCM8
hsa-miR-3120-5p	MCM8
hsa-miR-4279	MCM8
hsa-miR-1976	MCM8
hsa-miR-6747-3p	MCM8
hsa-miR-6727-3p	MCM8
hsa-miR-4722-3p	MCM8
hsa-miR-8062	MCM8
hsa-miR-5698	MCM8
hsa-miR-5698	MCM8
hsa-miR-744-3p	MCM8
hsa-miR-744-3p	MCM8
hsa-miR-4423-5p	MCM8
hsa-miR-4423-5p	MCM8
hsa-miR-6501-5p	MCM8
hsa-miR-6501-5p	MCM8
hsa-miR-143-5p	MCM8
hsa-miR-143-5p	MCM8
hsa-miR-4430	MCM8
hsa-miR-4430	MCM8
hsa-miR-3652	MCM8
hsa-miR-3652	MCM8
hsa-miR-504-3p	MCM8
hsa-miR-504-3p	MCM8
hsa-miR-3135b	MCM8
hsa-miR-3135b	MCM8
hsa-miR-6499-3p	MCM8
hsa-miR-6499-3p	MCM8
hsa-miR-367-5p	MCM8
hsa-miR-367-5p	MCM8
hsa-miR-1267	MCM8
hsa-miR-1267	MCM8
hsa-miR-4465	MCM8
hsa-miR-26b-5p	MCM8
hsa-miR-26a-5p	MCM8
hsa-miR-1297	MCM8
hsa-miR-6513-3p	MCM8
hsa-miR-4532	MCM8
hsa-miR-1247-3p	MCM8
hsa-miR-544a	MCM8

hsa-miR-4277	MCM8
hsa-miR-20a-3p	MCM8
hsa-miR-584-3p	MCM8
hsa-miR-4762-5p	MCM8
hsa-miR-1285-5p	MCM8
hsa-miR-6874-3p	MCM8
hsa-miR-148b-5p	MCM8
hsa-miR-216a-5p	MCM8
hsa-miR-122-5p	MCM8
hsa-miR-1304-3p	MCM8
hsa-miR-3664-5p	MCM8
hsa-miR-4639-3p	MCM8
hsa-miR-6794-3p	MCM8
hsa-miR-144-3p	MCM8
hsa-miR-16-1-3p	MCM8
hsa-miR-3606-3p	MCM8
hsa-miR-3653-3p	MCM8
hsa-miR-3658	MCM8
hsa-miR-513a-3p	MCM8
hsa-miR-513c-3p	MCM8
hsa-miR-5680	MCM8
hsa-miR-7856-5p	MCM8
hsa-miR-320b	TIMELESS
hsa-miR-877-3p	TIMELESS
hsa-miR-615-3p	TIMELESS
hsa-miR-484	TIMELESS
hsa-miR-324-5p	TIMELESS
hsa-miR-183-5p	TIMELESS
hsa-miR-548s	TIMELESS
hsa-miR-548s	TIMELESS
hsa-miR-3926	TIMELESS
hsa-miR-3926	TIMELESS
hsa-miR-3190-5p	TIMELESS
hsa-miR-3190-5p	TIMELESS
hsa-miR-5699-3p	TIMELESS
hsa-miR-5699-3p	TIMELESS
hsa-miR-4421	TIMELESS
hsa-miR-4421	TIMELESS
hsa-miR-6849-3p	TIMELESS
hsa-miR-6849-3p	TIMELESS
hsa-miR-6748-3p	TIMELESS
hsa-miR-6748-3p	TIMELESS
hsa-miR-3149	TIMELESS
hsa-miR-3591-5p	TIMELESS
hsa-miR-5089-5p	TIMELESS
hsa-miR-620	TIMELESS
hsa-miR-1270	TIMELESS
hsa-miR-4460	TIMELESS
hsa-miR-4727-3p	TIMELESS

hsa-miR-3167	TIMELESS
hsa-miR-876-5p	TIMELESS
hsa-miR-4683	TIMELESS
hsa-miR-1273f	TIMELESS
hsa-miR-6849-5p	TIMELESS
hsa-miR-3681-5p	TIMELESS
hsa-miR-6839-3p	TIMELESS
hsa-miR-4507	TIMELESS
hsa-miR-3940-5p	TIMELESS
hsa-miR-5001-3p	TIMELESS
hsa-miR-23b-5p	TIMELESS
hsa-miR-23a-5p	TIMELESS
hsa-miR-6781-3p	TIMELESS
hsa-miR-2682-3p	TIMELESS
hsa-miR-6774-5p	TIMELESS
hsa-miR-635	TIMELESS
hsa-miR-4772-3p	TIMELESS
hsa-miR-1304-3p	TIMELESS
hsa-miR-6890-3p	TIMELESS
hsa-miR-7703	TIMELESS
hsa-miR-6720-5p	TIMELESS
hsa-miR-6512-3p	TIMELESS
hsa-miR-4252	TIMELESS
hsa-miR-4793-3p	TIMELESS
hsa-miR-766-3p	TIMELESS
hsa-miR-1273g-3p	TIMELESS
hsa-miR-3940-3p	TIMELESS
hsa-miR-508-5p	TIMELESS
hsa-miR-1273e	TIMELESS
hsa-miR-1827	TIMELESS
hsa-miR-219a-2-3p	TIMELESS
hsa-miR-3664-3p	TIMELESS
hsa-miR-4649-3p	TIMELESS
hsa-miR-4695-5p	TIMELESS
hsa-miR-4722-5p	TIMELESS
hsa-miR-510-5p	TIMELESS
hsa-miR-512-5p	TIMELESS
hsa-miR-6808-5p	TIMELESS
hsa-miR-6893-5p	TIMELESS
hsa-miR-940	TIMELESS
hsa-miR-192-5p	CDC7
hsa-miR-215-5p	CDC7
hsa-miR-101-3p	CDC7
hsa-miR-615-3p	CDC7
hsa-let-7a-5p	CDC7
hsa-miR-30a-5p	CDC7
hsa-miR-30c-5p	CDC7
hsa-miR-30d-5p	CDC7
hsa-miR-30b-5p	CDC7

hsa-miR-30e-5p	CDC7
hsa-miR-29a-3p	CDC7
hsa-miR-26a-5p	CDC6
hsa-miR-26a-5p	CDC6
hsa-miR-886-3p	CDC6
hsa-miR-193b-3p	CDC6
hsa-miR-142-3p	CDC6
hsa-miR-361-3p	CDC6
hsa-miR-615-3p	CDC6
hsa-miR-501-5p	CDC6
hsa-miR-675-5p	CDC6
hsa-miR-6818-3p	CDC6
hsa-miR-4684-5p	CDC6
hsa-miR-6895-3p	CDC6
hsa-miR-593-3p	CDC6
hsa-miR-5699-3p	CDC6
hsa-miR-4421	CDC6
hsa-miR-339-5p	CDC6
hsa-miR-10b-5p	CDC6
hsa-miR-10a-5p	CDC6
hsa-miR-6732-3p	CDC6
hsa-miR-548ay-3p	CDC6
hsa-miR-548at-3p	CDC6
hsa-miR-548as-3p	CDC6
hsa-miR-548t-3p	CDC6
hsa-miR-548ap-3p	CDC6
hsa-miR-548aa	CDC6
hsa-miR-548c-3p	CDC6
hsa-miR-32-5p	CDC6
hsa-miR-92b-3p	CDC6
hsa-miR-92a-3p	CDC6
hsa-miR-193b-3p	POLE2
hsa-miR-24-3p	CDKN2A
hsa-let-7g-5p	CDKN2A
hsa-miR-125b-5p	CDKN2A
hsa-miR-125b-5p	CDKN2A
hsa-miR-492	CDKN2A
hsa-miR-10b-5p	CDKN2A
hsa-miR-10b-5p	CDKN2A
hsa-miR-155-5p	CDKN2A
hsa-miR-124-3p	CDKN2A
hsa-miR-215-5p	CDKN2A
hsa-miR-34a-5p	CDKN2A
hsa-miR-192-5p	CDKN2A
hsa-miR-16-5p	CDKN2A
hsa-miR-455-3p	CDKN2A
hsa-miR-423-5p	CDKN2A
hsa-miR-296-3p	CDKN2A
hsa-miR-615-3p	CDKN2A

hsa-miR-320a	CDKN2A
hsa-miR-193b-3p	POLA1
hsa-miR-124-3p	POLA1
hsa-miR-26b-5p	POLA1
hsa-miR-6885-3p	POLA1
hsa-miR-6882-3p	POLA1
hsa-miR-98-5p	DBF4
hsa-miR-30a-5p	DBF4
hsa-miR-30c-5p	DBF4
hsa-miR-30d-5p	DBF4
hsa-miR-30b-5p	DBF4
hsa-miR-30e-5p	DBF4
hsa-miR-8057	DBF4
hsa-miR-4325	DBF4
hsa-miR-24-3p	DBF4
hsa-miR-4284	DBF4
hsa-miR-4793-3p	DBF4
hsa-miR-4772-3p	DBF4
hsa-miR-1304-3p	DBF4
hsa-miR-6890-3p	DBF4
hsa-miR-615-3p	TONSL
hsa-miR-320a	TONSL
hsa-miR-92a-3p	TONSL
hsa-miR-365b-5p	TONSL
hsa-miR-365a-5p	TONSL
hsa-miR-8052	TONSL
hsa-miR-3199	TONSL
hsa-miR-6747-3p	TONSL
hsa-miR-4722-3p	TONSL
hsa-miR-6727-3p	TONSL
hsa-miR-1203	TONSL
hsa-miR-7157-3p	ING5
hsa-miR-6737-3p	ING5
hsa-miR-5008-3p	ING5
hsa-miR-1914-5p	ING5
hsa-miR-8485	ING5
hsa-miR-649	ING5
hsa-miR-6515-5p	ING5
hsa-miR-4443	ING5
hsa-miR-6827-5p	ING5
hsa-miR-6797-5p	ING5
hsa-miR-1249-5p	ING5
hsa-miR-6133	ING5
hsa-miR-6130	ING5
hsa-miR-6129	ING5
hsa-miR-6127	ING5
hsa-miR-4510	ING5
hsa-miR-4419a	ING5
hsa-miR-1225-3p	ING5

hsa-miR-432-5p	ING5
hsa-miR-6756-3p	ING5
hsa-miR-3127-3p	ING5
hsa-miR-4650-5p	ING5
hsa-miR-1972	ING5
hsa-miR-6769a-3p	ING5
hsa-miR-331-3p	ING5
hsa-miR-193a-5p	ING5
