# Supplementary material for: Application of Protein-Protein Interaction Network Analysis in Order to Identify Cervical Cancer miRNA and mRNA Biomarkers
Source: ScientificWorldJournal. 2023 Sep 14;2023:6626279. doi: 10.1155/2023/6626279 (PMC10513823; doi:10.1155/2023/6626279)
Supplement: Supplementary Materials — This paper includes four supplementary files named S1 to S4. [file 6626279.f1.zip › Supplementary file S4 (1).pdf]

Gene Ontology- Biological process

description	pval adj	genes
Activation of the pre-replicative complex	4.61E-22	CDC45, POLE2, MCM6, MCM2, CDC7, DBF4, CDC6, POLA1, MCM4, MCM10
Mitotic G1 phase and G1/S transition	1.19E-17	CDC45, POLE2, MCM6, MCM2, CDC7, DBF4, CDKN2A, CDC6, POLA1, MCM4, MCM10
Activation of ATR in response to replication stress	3.93E-16	CDC45, MCM6, MCM2, CDC7, DBF4, CDC6, MCM4, MCM10
G1/S Transition	4.28E-16	CDC45, POLE2, MCM6, MCM2, CDC7, DBF4, CDC6, POLA1, MCM4, MCM10
DNA Replication Pre-Initiation	1.07E-15	CDC45, POLE2, MCM6, MCM2, CDC7, DBF4, CDC6, POLA1, MCM4, MCM10
DNA Replication	1.29E-14	CDC45, POLE2, MCM6, MCM2, CDC7, DBF4, CDC6, POLA1, MCM4, MCM10
Cell Cycle, Mitotic	9.69E-12	CDC45, POLE2, MCM6, MCM2, CDC7, DBF4, CDKN2A, CDC6, POLA1, MCM4, MCM10
G2/M Checkpoints	5.03E-11	CDC45, MCM6, MCM2, CDC7, DBF4, CDC6, MCM4, MCM10
Cell Cycle Checkpoints	6.69E-11	CDC45, MCM6, MCM2, CDC7, DBF4, CDKN2A, CDC6, MCM4, MCM10
Cell Cycle	6.89E-11	CDC45, POLE2, MCM6, MCM2, CDC7, DBF4, CDKN2A, CDC6, POLA1, MCM4, MCM10
Synthesis of DNA	3.12E-10	CDC45, POLE2, MCM6, MCM2, CDC6, POLA1, MCM4
DNA strand elongation	1.88E-09	CDC45, MCM6, MCM2, POLA1, MCM4
S Phase	2.28E-09	CDC45, POLE2, MCM6, MCM2, CDC6, POLA1, MCM4
Unwinding of DNA	3.68E-09	CDC45, MCM6, MCM2, MCM4
Orc1 removal from chromatin	8.64E-06	MCM6, MCM2, CDC6, MCM4
Switching of origins to a post-replicative state	2.26E-05	MCM6, MCM2, CDC6, MCM4
G1/S-Specific Transcription	3.11E-05	CDC45, CDC6, POLA1
Assembly of the pre-replicative complex	8.26E-05	MCM6, MCM2, CDC6, MCM4
DNA replication initiation	0.000229	POLE2, POLA1
Evasion of Oxidative Stress Induced Senescence Due to p14ARF Defects	0.006499	CDKN2A
Evasion of Oncogene Induced Senescence Due to p14ARF Defects	0.006499	CDKN2A
Evasion of Oncogene Induced Senescence Due to Defective p16INK4A binding to CDK4	0.011367	CDKN2A
Evasion of Oxidative Stress Induced Senescence Due to Defective p16INK4A binding to CDK4	0.011367	CDKN2A
Defective Intrinsic Pathway for Apoptosis Due to p14ARF Loss of Function	0.011367	CDKN2A
Diseases of cellular response to stress	0.013632	CDKN2A
Evasion of Oxidative Stress Induced Senescence Due to p16INK4A Defects	0.013632	CDKN2A
Evasion of Oncogene Induced Senescence Due to p16INK4A Defects	0.013632	CDKN2A
Evasion of Oncogene Induced Senescence Due to Defective p16INK4A binding to CDK4 and CDK6	0.013632	CDKN2A
Diseases of Cellular Senescence	0.013632	CDKN2A
Evasion of Oxidative Stress Induced Senescence Due to Defective p16INK4A binding to CDK4 and CDK6	0.013632	CDKN2A
Diseases of programmed cell death	0.02376	CDKN2A, POLA1
HDR through Homologous Recombination (HRR) or Single Strand Annealing (SSA)	0.037452	POLE2, TIMELESS
Homology Directed Repair	0.039594	POLE2, TIMELESS
CDC6 association with the ORC:origin complex	0.039934	CDC6
DNA Double-Strand Break Repair	0.046256	POLE2, TIMELESS
Leading Strand Synthesis	0.046256	POLA1
Polymerase switching	0.046256	POLA1
Inhibition of replication initiation of damaged DNA by RB1/E2F1	0.046256	POLA1
Regulation of TP53 Activity	0.046256	CDKN2A, ING5
Telomere C-strand synthesis initiation	0.046256	POLA1
Removal of the Flap Intermediate	0.046256	POLA1
Processive synthesis on the lagging strand	0.048353	POLA1

Gene Ontology- Cellular Component

description	pval adj	genes
MCM complex	4.03E-12	MCM6, TONSL, MCM2, MCM8, MCM4
nucleoplasm	1.65E-10	CDC45, POLE2, MCM6, TONSL, WDHD1, MCM2, MCM8, TIMELESS, CDC7, DBF4, CDKN2A, CDC6, POLA1, ING5, MCM4, MCM10
CMG complex	1.51E-09	CDC45, MCM6, MCM2, MCM4
nucleus	5.40E-07	CDC45, POLE2, MCM6, TONSL, WDHD1, MCM2, MCM8, TIMELESS, CDC7, DBF4, CDKN2A, CDC6, POLA1, ING5, MCM4, MCM10
replication fork protection complex	1.61E-05	TIMELESS, MCM10
chromosome	3.93E-05	MCM6, MCM2, MCM8, TIMELESS, ING5, MCM4
nuclear replication fork	0.000253	TONSL, WDHD1
nuclear body	0.000698	POLE2, TONSL, DBF4, CDKN2A
chromosome, telomeric region	0.001609	MCM6, MCM2, MCM4
nucleolus	0.004615	MCM2, CDKN2A, CDC6, POLA1, MCM10
intercellular bridge	0.005148	CDC7, CDC6
Dbf4-dependent protein kinase complex	0.005148	DBF4
DNA replication preinitiation complex	0.005148	CDC45
MCM8-MCM9 complex	0.005148	MCM8
senescence-associated heterochromatin focus	0.008463	CDKN2A
FACT complex	0.008463	TONSL
alpha DNA polymerase:primase complex	0.008776	POLA1
delta DNA polymerase complex	0.008776	POLA1
epsilon DNA polymerase complex	0.008776	POLE2
mitotic spindle	0.008776	CDC7, CDC6
nuclear origin of replication recognition complex	0.013821	MCM2
MOZ/MORF histone acetyltransferase complex	0.013821	ING5
DNA replication factor A complex	0.019066	TONSL
spindle midzone	0.03497	CDC6
histone acetyltransferase complex	0.044174	ING5

Gene Ontology- Molecular Function

description	pval adj	genes
DNA replication origin binding	5.88E-11	CDC45, MCM2, CDC6, POLA1, MCM10
single-stranded DNA binding	6.44E-11	CDC45, MCM6, MCM2, MCM8, POLA1, MCM4, MCM10
single-stranded DNA helicase activity	4.05E-08	MCM6, MCM2, MCM8, MCM4
DNA helicase activity	2.96E-06	MCM6, MCM2, MCM8, MCM4
DNA binding	3.07E-05	POLE2, MCM6, WDHD1, MCM2, MCM8, TIMELESS, CDKN2A, POLA1, MCM4, MCM10
helicase activity	5.30E-05	MCM6, MCM2, MCM8, MCM4
chromatin binding	0.000277	CDC45, WDHD1, MCM8, POLA1, ING5
ATP hydrolysis activity	0.001082	MCM6, MCM2, MCM8, MCM4
DNA-directed DNA polymerase activity	0.001125	POLE2, POLA1
nucleotide binding	0.002492	MCM6, MCM2, MCM8, CDC7, CDC6, POLA1, MCM4
ATP binding	0.005415	MCM6, MCM2, MCM8, CDC7, CDC6, MCM4
ubiquitin-protein transferase inhibitor activity	0.006909	CDKN2A
pyrimidine nucleotide binding	0.006909	POLA1
single-stranded 3'-5' DNA helicase activity	0.008417	MCM6
MutLbeta complex binding	0.008417	MCM8
MutSbeta complex binding	0.008417	MCM8
enzyme binding	0.013311	MCM2, MCM8, MCM10
protein binding	0.015676	CDC45, POLE2, MCM6, TONSL, WDHD1, MCM2, MCM8, TIMELESS, CDC7, DBF4, CDKN2A, CDC6, POLA1, ING5, MCM4, MCM10
MutSalpha complex binding	0.015686	MCM8
purine nucleotide binding	0.015686	POLA1
ubiquitin ligase inhibitor activity	0.019192	CDKN2A
histone binding	0.022363	TONSL, MCM2
cyclin-dependent protein serine/threonine kinase inhibitor activity	0.022363	CDKN2A
MDM2/MDM4 family protein binding	0.022363	CDKN2A
3'-5' DNA helicase activity	0.023248	MCM2
SUMO transferase activity	0.035991	CDKN2A

Reactome pathways

description	pval adj	genes
Activation of the pre-replicative complex	4.61E-22	CDC45, POLE2, MCM6, MCM2, CDC7, DBF4, CDC6, POLA1, MCM4, MCM10
Mitotic G1 phase and G1/S transition	1.19E-17	CDC45, POLE2, MCM6, MCM2, CDC7, DBF4, CDKN2A, CDC6, POLA1, MCM4, MCM10
Activation of ATR in response to replication stress	3.93E-16	CDC45, MCM6, MCM2, CDC7, DBF4, CDC6, MCM4, MCM10
G1/S Transition	4.28E-16	CDC45, POLE2, MCM6, MCM2, CDC7, DBF4, CDC6, POLA1, MCM4, MCM10
DNA Replication Pre-Initiation	1.07E-15	CDC45, POLE2, MCM6, MCM2, CDC7, DBF4, CDC6, POLA1, MCM4, MCM10
DNA Replication	1.29E-14	CDC45, POLE2, MCM6, MCM2, CDC7, DBF4, CDC6, POLA1, MCM4, MCM10
Cell Cycle, Mitotic	9.69E-12	CDC45, POLE2, MCM6, MCM2, CDC7, DBF4, CDKN2A, CDC6, POLA1, MCM4, MCM10

G2/M Checkpoints	5.03E-11	CDC45, MCM6, MCM2, CDC7, DBF4, CDC6, MCM4, MCM10
Cell Cycle Checkpoints	6.69E-11	CDC45, MCM6, MCM2, CDC7, DBF4, CDKN2A, CDC6, MCM4, MCM10
Cell Cycle	6.89E-11	CDC45, POLE2, MCM6, MCM2, CDC7, DBF4, CDKN2A, CDC6, POLA1, MCM4, MCM10
Synthesis of DNA	3.12E-10	CDC45, POLE2, MCM6, MCM2, CDC6, POLA1, MCM4
DNA strand elongation	1.88E-09	CDC45, MCM6, MCM2, POLA1, MCM4
S Phase	2.28E-09	CDC45, POLE2, MCM6, MCM2, CDC6, POLA1, MCM4
Unwinding of DNA	3.68E-09	CDC45, MCM6, MCM2, MCM4
Orc1 removal from chromatin	8.64E-06	MCM6, MCM2, CDC6, MCM4
Switching of origins to a post-replicative state	2.26E-05	MCM6, MCM2, CDC6, MCM4
G1/S-Specific Transcription	3.11E-05	CDC45, CDC6, POLA1
Assembly of the pre-replicative complex	8.26E-05	MCM6, MCM2, CDC6, MCM4
DNA replication initiation	0.000229	POLE2, POLA1
Evasion of Oxidative Stress Induced Senescence Due to p14ARF Defects	0.006499	CDKN2A
Evasion of Oncogene Induced Senescence Due to p14ARF Defects	0.006499	CDKN2A
Evasion of Oncogene Induced Senescence Due to Defective p16INK4A binding to CDK4	0.011367	CDKN2A
Evasion of Oxidative Stress Induced Senescence Due to Defective p16INK4A binding to CDK4	0.011367	CDKN2A
Defective Intrinsic Pathway for Apoptosis Due to p14ARF Loss of Function	0.011367	CDKN2A
Diseases of cellular response to stress	0.013632	CDKN2A
Evasion of Oxidative Stress Induced Senescence Due to p16INK4A Defects	0.013632	CDKN2A
Evasion of Oncogene Induced Senescence Due to p16INK4A Defects	0.013632	CDKN2A
Evasion of Oncogene Induced Senescence Due to Defective p16INK4A binding to CDK4 and CDK6	0.013632	CDKN2A
Diseases of Cellular Senescence	0.013632	CDKN2A
Evasion of Oxidative Stress Induced Senescence Due to Defective p16INK4A binding to CDK4 and CDK6	0.013632	CDKN2A
Diseases of programmed cell death	0.02376	CDKN2A, POLA1
HDR through Homologous Recombination (HRR) or Single Strand Annealing (SSA)	0.037452	POLE2, TIMELESS
Homology Directed Repair	0.039594	POLE2, TIMELESS
CDC6 association with the ORC:origin complex	0.039934	CDC6
DNA Double-Strand Break Repair	0.046256	POLE2, TIMELESS
Leading Strand Synthesis	0.046256	POLA1
Polymerase switching	0.046256	POLA1
Inhibition of replication initiation of damaged DNA by RB1/E2F1	0.046256	POLA1
Regulation of TP53 Activity	0.046256	CDKN2A, ING5
Telomere C-strand synthesis initiation	0.046256	POLA1
Removal of the Flap Intermediate	0.046256	POLA1
Processive synthesis on the lagging strand	0.048353	POLA1

miRNA family

Term	FDR	miRNA
mir-30 family	3.33E-04	hsa-mir-30a,hsa-mir-30b,hsa-mir-30c-1,hsa-mir-30c-2,hsa-mir-30d,hsa-mir-30e
mir-10 family	0.0274	hsa-mir-10a,hsa-mir-10b,hsa-mir-125b-1,hsa-mir-125b-2,hsa-mir-99a
mir-124 family	0.032	hsa-mir-124-1,hsa-mir-124-2,hsa-mir-124-3
mir-26 family	0.032	hsa-mir-26a-1,hsa-mir-26a-2,hsa-mir-26b
mir-290 family	0.032	hsa-mir-371a,hsa-mir-371b,hsa-mir-372
let-7 family	0.0379	hsa-let-7a-1,hsa-let-7a-2,hsa-let-7a-3,hsa-let-7b,hsa-let-7g,hsa-mir-98
